# Changes in DNA methylation hallmark alterations in chromatin accessibility and gene expression for eye lens differentiation

**DOI:** 10.1186/s13072-022-00440-z

**Published:** 2022-03-05

**Authors:** Joshua Disatham, Lisa Brennan, Xiaodong Jiao, Zhiwei Ma, J. Fielding Hejtmancik, Marc Kantorow

**Affiliations:** 1 Department of Biomedical Science, Charles E. Schmidt College of Medicine, Florida Atlantic University, Boca Raton, FL USA; 2 Ophthalmic Genetics and Visual Function Branch, National Eye Institute, National Institutes of Health, Bethesda, MD USA

**Keywords:** DNA methylation, Bisulfite sequencing, RNA-seq, ATAC-seq, Lens, Differentiation, Gene regulation, Chromatin, Transcriptional regulation

## Abstract

**Background:**

Methylation at cytosines (mCG) is a well-known regulator of gene expression, but its requirements for cellular differentiation have yet to be fully elucidated. A well-studied cellular differentiation model system is the eye lens, consisting of a single anterior layer of epithelial cells that migrate laterally and differentiate into a core of fiber cells. Here, we explore the genome-wide relationships between mCG methylation, chromatin accessibility and gene expression during differentiation of eye lens epithelial cells into fiber cells.

**Results:**

Whole genome bisulfite sequencing identified 7621 genomic loci exhibiting significant differences in mCG levels between lens epithelial and fiber cells. Changes in mCG levels were inversely correlated with the differentiation state-specific expression of 1285 genes preferentially expressed in either lens fiber or lens epithelial cells (Pearson correlation *r* = − 0.37, *p* < 1 × 10^–42^). mCG levels were inversely correlated with chromatin accessibility determined by assay for transposase-accessible sequencing (ATAC-seq) (Pearson correlation *r* = − 0.86, *p* < 1 × 10^–300^). Many of the genes exhibiting altered regions of DNA methylation, chromatin accessibility and gene expression levels in fiber cells relative to epithelial cells are associated with lens fiber cell structure, homeostasis and transparency. These include lens crystallins (CRYBA4, CRYBB1, CRYGN, CRYBB2), lens beaded filament proteins (BFSP1, BFSP2), transcription factors (HSF4, SOX2, HIF1A), and Notch signaling pathway members (NOTCH1, NOTCH2, HEY1, HES5). Analysis of regions exhibiting cell-type specific alterations in DNA methylation revealed an overrepresentation of consensus sequences of multiple transcription factors known to play key roles in lens cell differentiation including HIF1A, SOX2, and the MAF family of transcription factors.

**Conclusions:**

Collectively, these results link DNA methylation with control of chromatin accessibility and gene expression changes required for eye lens differentiation. The results also point to a role for DNA methylation in the regulation of transcription factors previously identified to be important for lens cell differentiation.

**Supplementary Information:**

The online version contains supplementary material available at 10.1186/s13072-022-00440-z.

## Background

It is well-accepted that epigenetic mechanisms can regulate cellular differentiation pathways required for the form and function of mature tissues [1–3]. Many of these pathways culminate in chromatin accessibility changes that modulate the ability of required transcription factors (TFs) to activate or repress the expression of specific genes through altered access and binding to key cis-regulatory sequences [4–6].

A major epigenetic mechanism regulating gene expression is DNA methylation at cytosine residues to produce methyl cytosine (mC). DNA methylation at cytosine residues occurs at CG, CHG, and CHH nucleotides (where H represents A, T, or C). Production of mC is dependent on the activities of DNA methyltransferases (DNMTs) that require S-adenosylmethionine that acts as a methyl donor [7–10]. Increased levels of DNA methylation are associated with nucleosome compaction and reduced accessibility of TFs to their cognate binding regions resulting in altered gene expression [8, 11–13]. In addition, the presence of mCG in TF binding sites can alter their DNA binding affinities [8, 14–18] to modulate gene expression. Consistent with these properties, DNA methylation has been shown to be important for the development, differentiation or maturation of embryonic stem cells [19], auditory epithelium [14], muscle [20, 21], adipocytes [22], trophoblasts [23], and chondrocytes [24].

Here, we examined the potential role of DNA methylation on the modulation of chromatin accessibility changes and the expression of key genes required for the differentiation of immature eye lens cells into mature transparent cells. A major feature of the lens is that, unlike many tissues, it grows both embryologically and throughout adult life through execution of a continuous cellular differentiation program resulting in two morphologically distinct cell populations [25]. The lens comprises an anterior surface monolayer of undifferentiated epithelial cells that, upon withdrawing from the cell cycle, differentiate into elongated, organelle-free lens fiber cells that make up the bulk of the lens, including undergoing karyolysis in the lens central nucleus [25–40]. These differentiation state-specific and morphologically distinct cellular populations can be isolated in quantities sufficient for molecular and biochemical analysis through microdissection [32, 35].

Lens cell differentiation is hallmarked by the expression of critical regulatory and structural genes [35, 41–43]. The differentiation state-specific expression patterns of these genes suggests that specific regulatory mechanisms operate to govern their expression levels during lens cell differentiation. Consistently, previous studies have identified multiple transcription factors [25, 28, 29, 41, 44–46] required for their differentiation state-specific expression patterns. Recently, epigenetic programming was shown to play an important role in the regulation of gene expression during lens fiber cell differentiation since ATAC (assay for transposase-accessible chromatin) sequencing of undifferentiated lens epithelial and differentiated fiber cells identified genome-wide changes in chromatin accessibility associated with the expression of multiple genes during lens differentiation [32, 47].

Here, we explored the role of epigenetic programming in control of lens cell differentiation by interrogating the differentiation state-specific relationship between DNA methylation, chromatin accessibility and gene expression levels between chicken lens epithelial and fiber cells using a combination of bisulfite sequencing, ATAC-seq and RNA-seq. Our analysis revealed that specific genomic DNA methylation patterns characterize the differentiation states of lens cells and that specific DNA methylation signatures correlate with differentiation state-specific changes in chromatin accessibility and gene expression levels. Examination of individual gene functions implicates DNA methylation as a factor in the regulation of multiple important lens functions including lens cell structure, homeostasis, and transparency. Analysis of DNA sequences with altered DNA methylation levels revealed the presence of consensus binding sequences for a wide variety of transcription factors known to regulate lens differentiation state-specific gene expression.

Collectively, these results establish DNA methylation as a key feature of lens cell differentiation and implicate specific DNA methylation signatures in the regulation of chromatin accessibility and gene expression. They also provide evidence that key DNA methylation patterns govern the binding, activity and availability of established transcription factors known to be required for lens differentiation. Finally, they provide a blueprint for studies aimed at identifying the role of DNA methylation in the differentiation of more complex tissues.

## Results

### Lens epithelial and fiber cells show differentiation state-specific patterns of genome-wide DNA methylation

**Fig. 1:**
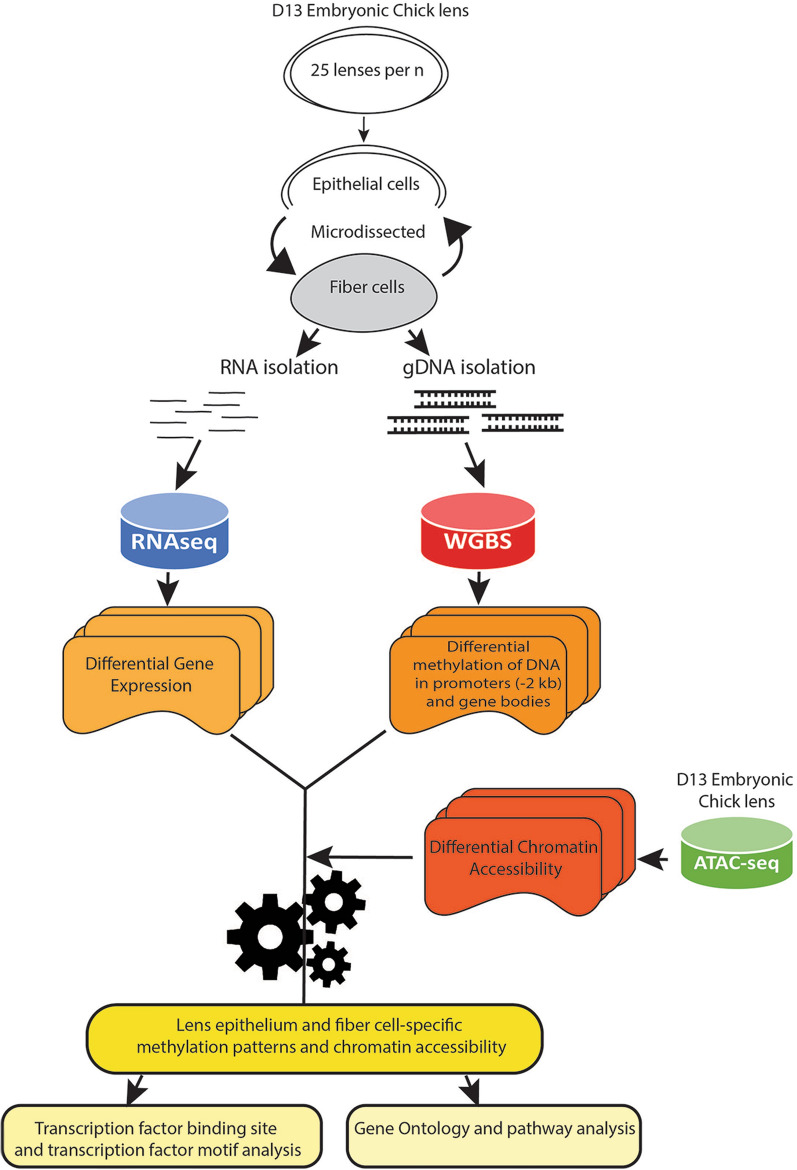
Multiomics analysis combines high-throughput sequencing techniques to elucidate novel features of lens differentiation. Workflow of the multiomics-based approach. Biological triplicate pools of 25 lenses from E13 embryonic chick were microdissected into undifferentiated lens epithelial cells and differentiated lens fiber cells. RNA isolated from samples was used for RNAseq to elucidate differentially expressed genes. Genomic DNA (gDNA) isolated from samples was used for whole genome bisulfite sequencing (WGBS) to elucidate differentially methylated genomic regions. ATACseq data from a previously published study on microdissected lenses from E13 embryonic chick [217] were used to identify changes in chromatin accessibility. Integrating these data reveal the range and spectrum of lens gene expression patterns characterized by specific methylation patterns and chromatin accessibility during lens cell differentiation. Transcription factor binding site (TFBS) and transcription factor (TF) motif analysis and gene ontology (GO) analysis was also performed

A combination of RNA-sequencing (RNAseq), whole genome bisulfite sequencing (WGBS) and chromatin accessibility (ATAC-seq) was employed to elucidate the potential role of DNA methylation in the regulation of chromatin structure and gene expression and thus lens cell differentiation (Fig. 1). 25 lenses per biological replicate from embryonic day 13 (E13) chick were microdissected into lens epithelial cell populations that comprised both the central and equatorial lens epithelia and lens fiber cell populations comprising both the peripheral and central lens fibers. The microdissected lens cell populations were subjected to genome-wide bisulfite sequencing. A total of 6 samples from two groups (three biological replicates of lens epithelial cells and three biological replicates of lens fiber cells) were examined yielding at least 125 million clean reads per sample with an average mapping rate of 83.54%. Pearson correlation analysis of the samples followed by dendrogram clustering confirmed that biological replicates from the same groups are more strongly correlated than replicates from different groups (Additional file 1: Figure S1A, B, lens epithelial cells Pearson’s *r* = 0.965–0.972, lens fiber cells Pearson’s *r* = 0.967–0.976, lens fiber cells to epithelial cells Pearson’s *r* = 0.941–0.963). These results confirm the reliability and reproducibility of the bisulfite sequencing data.

**Fig. 2:**
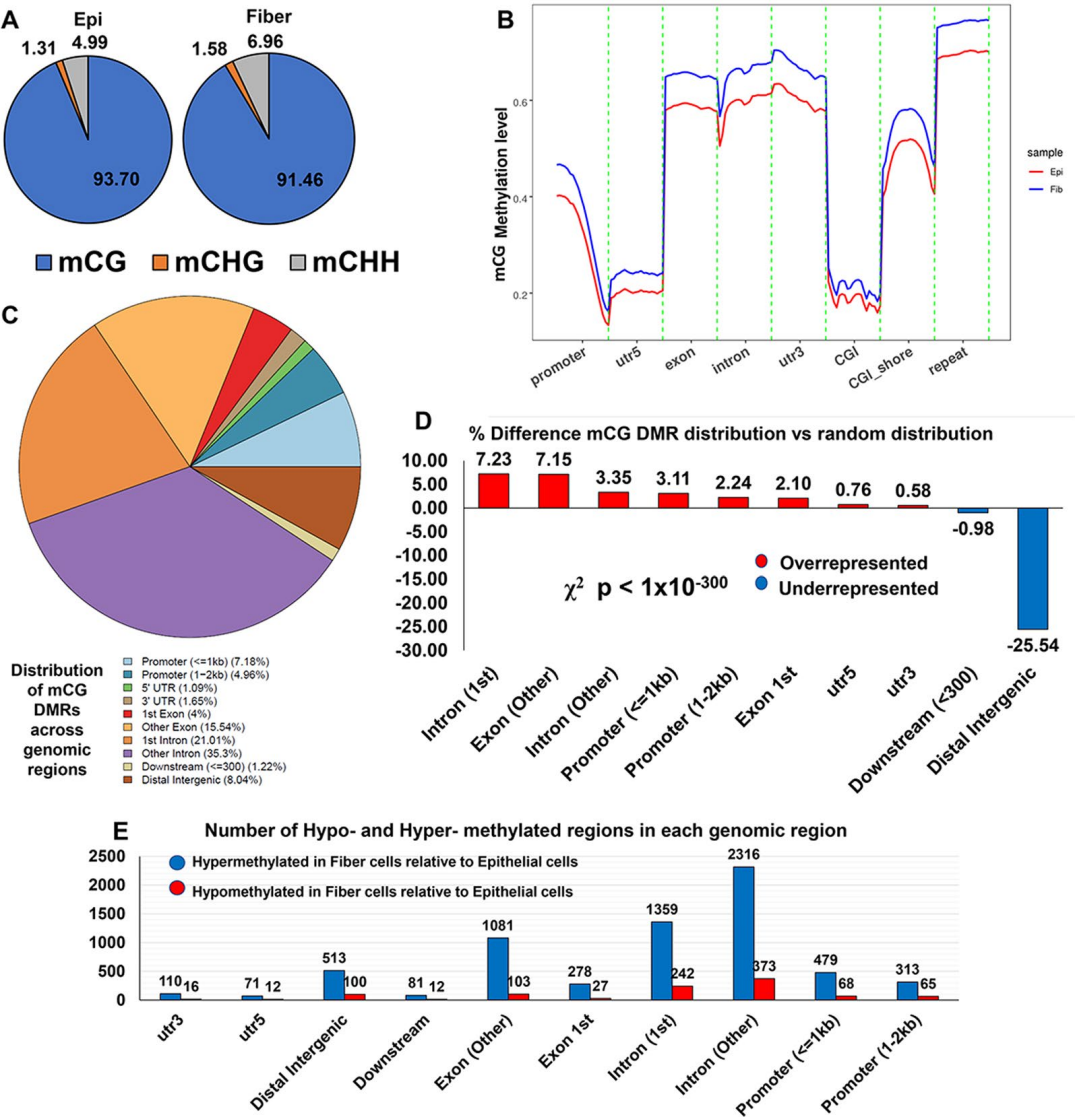
Differentiation-state-specific patterns of genome-wide DNA methylation are detected between lens epithelial and fiber cells. **A** Proportion of methylcytosines (mCs) that are mCG, mCHG, or mCHH found in lens epithelial and fiber cells. **B** mCG methylation levels at indicated genomic regions in lens epithelial and fiber cells. **C** Distribution of mCG differentially methylated regions (DMRs) at indicated genomic regions. **D** Percentage distribution of mCG DMRs at indicated genomic regions relative to random distribution across the genome. Positive values indicate that mCG DMRs cluster in the indicated genomic region are in excess of what is expected due to a random distribution while negative values indicate that mCG DMRs cluster in the indicated genomic region are less than what is expected due to a random distribution. **E** Number of hypo- and hyper-methylated regions at indicated genomic regions. Hypomethylated regions are demethylated in lens fiber cells compared to lens epithelial cells while hypermethylated regions are more methylated in lens fiber cells compared to lens epithelial cells

Genome-wide DNA methylation patterns detected in lens epithelial cells and fiber cells were mapped and quantified. Of the 446 million cytosines in the embryonic chick lens genome, between 3.4 and 4% were methylated in lens epithelial or fiber cells, with a large majority of methylated cytosines (> 91%) being present in mCG dinucleotide pairs (Fig. 2A, Additional file 5: Table S1). This result is consistent with previous studies in other vertebrate animal model systems and tissues that also exhibit a greater proportion of mCG [14, 48]. Subsequent examination of the global methylation levels (ratio of mCG/CG) across different genomic regions revealed that fiber cells exhibited a higher overall degree of methylation than epithelial cells at presumptive promoter regions (2 kb upstream of gene transcription start sites), gene bodies (utr5, exon, intron, utr3), CpG islands in intergenic regions (CGI) and their immediate 2-kb flanking regions (CGI shore), and repeat regions (low complexity DNA regions) (Fig. 2B, Additional file 1: Figure S1C).

Differentially methylated regions (DMRs) are established regulators of major gene expression changes [8, 49, 50]. Our analysis identified 7621 total mCG DMRs across the E13 embryonic chick lens genome. These 7621 regions contained differentiation state-specific differences in mCG methylation levels in lens epithelial cells compared to lens fiber cells. A majority of these regions (78.59%) were mapped to gene bodies (utr5, utr3, 1st exon, 1st intron, other exon, other intron) while 12.14% mapped to presumptive promoter regions (within 2 kb upstream from transcription start site). A minority were mapped to intergenic regions (8.04%) (Fig. 2C). To determine whether the observed distribution of mCG DMRs could have occurred by chance, we compared the distribution patterns of differentially methylated regions between lens epithelial and fiber cells to a computer-generated random distribution of differentially methylated regions across the genome. We found that 7621 mCG DMRs clustered more in genebody and promoter regions and less in proximal downstream and distal intergenic regions than what would be expected if the distribution were due to random chance (Fig. 2D, Chi-square *p* < 1 × 10^–300^). Of the 7621 identified mCG differentially methylated regions, a large majority (6601) were hypermethylated in fiber cells compared to epithelial cells, while a smaller group (1020) were hypomethylated in fiber cells compared to epithelial cells (Fig. 2E, Additional file 6: Table S2). Classifying the mCG DMRs based on genomic region also revealed that most genomic regions containing mCG DMRs have increased methylation in fiber cells compared to epithelial cells. Interestingly, there are significant differences in the genomic distribution of hypermethylated regions versus the distribution of hypomethylated regions (Additional file 1: Figure S1D, Chi-square *p* < 1 × 10^–81^). Specifically, regions that were hypermethylated in fiber cells relative to epithelial cells tend to cluster more in exons and proximal promoter regions (≤ 1 kb upstream from gene transcription start sites) and regions that were hypomethylated in fiber cells relative to epithelial cells tend to cluster more in introns, intergenic, and distal promoter regions (1–2 kb upstream from gene transcription start sites). This asymmetric distribution of hypomethylated regions versus hypermethylated regions has also been reported in a previous study comparing DNA methylation patterns across tumor types [51]. A possible explanation for this asymmetric distribution is that certain regulatory elements in the proximal promoter (≤ 1 kb from TSS), and exons are required for epithelial cell gene expression that upon hypermethylation, lead to decreased expression in the fiber cells. In contrast certain regulatory elements found in the distal promoters (1–2 kb from TSS), regions might be required for fiber cell introns, and distal intergenic gene expression and are only active once these sites become hypomethylated.

Collectively, these data identify significant differences in DNA methylation patterns between lens epithelial and fiber cells and they suggest that changes in DNA methylation are a major feature of the lens differentiation program.

### RNA-sequencing reveals novel lens epithelial cell and fiber cell gene expression patterns

As a first step towards establishing the role of DNA methylation in lens cell differentiation, levels of DNA methylation were correlated with specific gene expression changes occurring during lens cell differentiation as established by RNA-seq of microdissected lens epithelia and fiber cells.

**Fig. 3:**
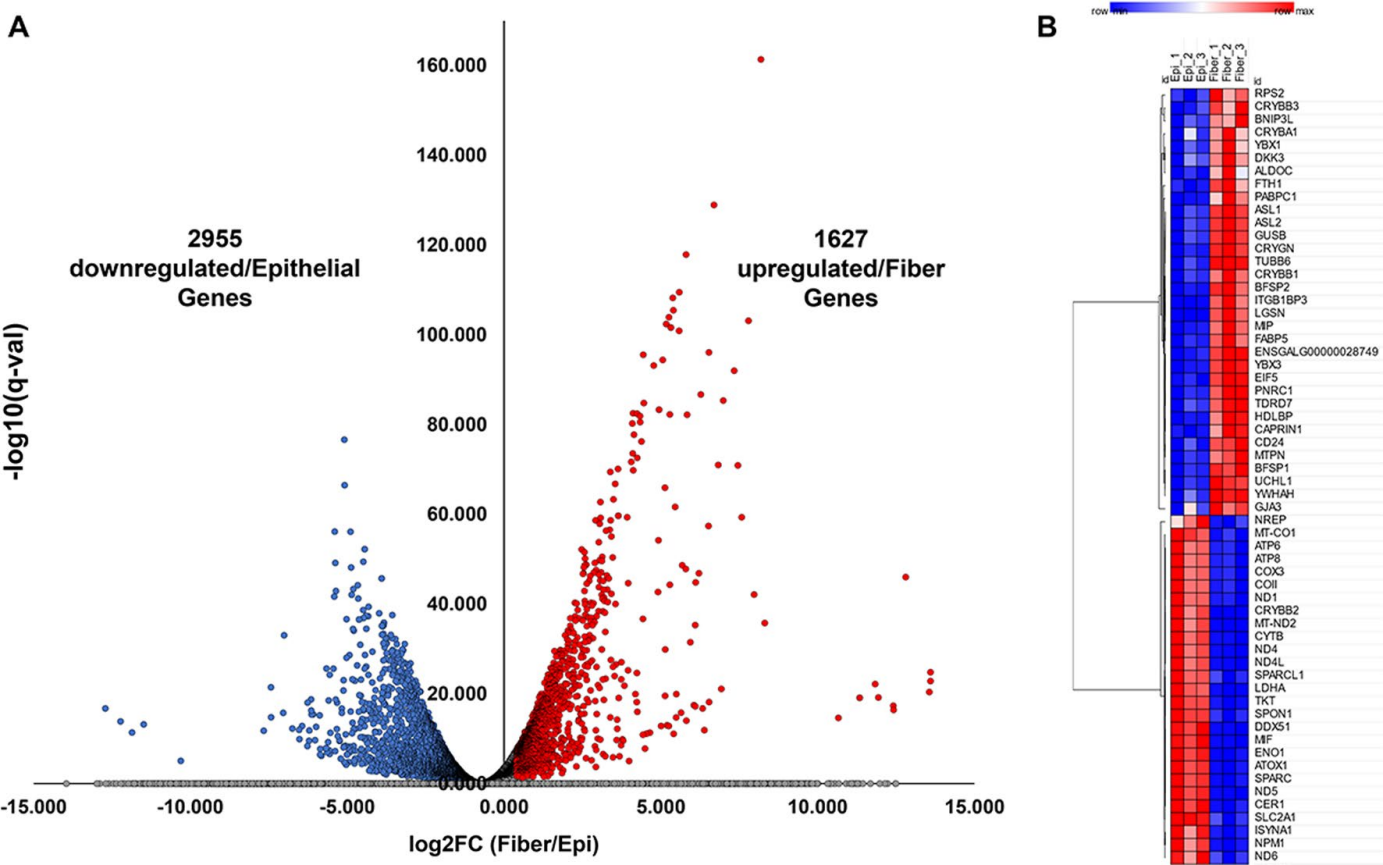
RNA-sequencing reveals novel lens epithelial cell and fiber cell gene expression patterns. **A** Volcano plot showing differentially expressed genes (DEGs) between lens fiber cells compared to lens epithelial cells (log2FC > 0.4 or  < − 0.4 and adjusted *p* < 0.05). **B** Top 60 DEGs (ranked by lowest adjusted *p* value) between lens fiber cells compared to lens epithelial cells

Twenty-five lenses per biological replicate from embryonic day 13 (E13) chick were microdissected into specific epithelial and fiber cell populations and RNAseq was conducted. One thousand six hundred and twenty-seven genes exhibited significantly higher levels of expression in fiber cells relative to epithelial cells (log2FC > 0.4, *q* < 0.05) and 2955 genes had significantly lower levels of expression in fiber cells compared to epithelial cells (log2FC < − 0.4, *q* < 0.05) (Fig. 3A, Additional file 7: Table S3). Consistent with their previously established expression patterns [35], the top 60 differentially expressed genes between lens epithelial and fiber cells (ranked by lowest adjusted *p* value) included crystallins (CRYBB3, CRYBA1, ASL1, ASL2, CRYGN, CRYBB1), beaded filament proteins (BFSP1, BFSP2), and the RNA binding protein (TDRD7) and connexin (GJA3) (Fig. 3B). An analysis of biological pathways specific to identified gene expression differences between these cell populations using Enrichr [52–54] identified a wide variety of significantly enriched biological processes and pathways associated with the top 200 epithelial cell genes (log2FC < − 0.4, *q* < 0.05, ranked by highest FPKM in epithelial cells to lowest) and the top 200 fiber cell genes (log2FC > 0.4, *q* < 0.05, ranked by highest FPKM in fiber cells to lowest) (Additional file 2: Figure S2A–D, Additional file 8: Table S4). The data identify that a majority of differentially expressed genes exhibit decreased expression in lens fiber cells (Fig. 3A) consistent with the overall increase in mCG DNA methylation levels in fiber cells during lens cell differentiation (Fig. 2B).

### Differentiation state-specific methylation patterns parallel differentiation state-specific gene expression changes during lens fiber cell differentiation

**Fig. 4:**
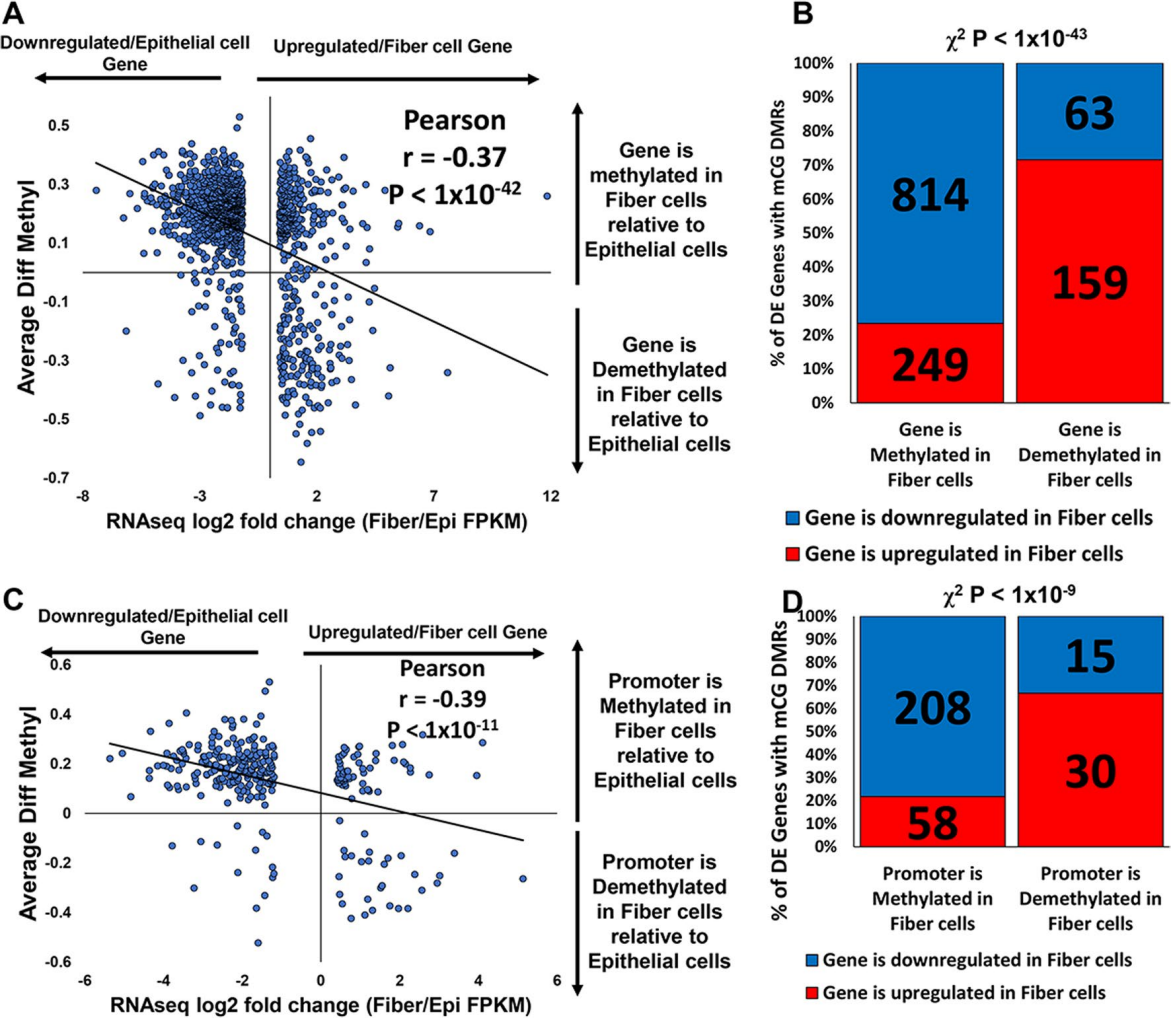
Differentiation-state-specific methylation patterns parallel differentiation state-specific gene expression changes during lens fiber cell differentiation. **A** Scatter plot of average change in mCG methylation (Average diff. Methyl) of all mCG DMRs mapped to the promoter (− 2 kb upstream of transcription start site) and genebody versus the log2-fold change in gene expression of differentially expressed genes (DEGs) between lens fiber cells and lens epithelial cells. Data indicate only DEGs that contain at least one mCG DMR. Pearson correlation *r* = − 0.37, *p* < 1 × 10^–42^. **B** Proportion of DEGs (upregulated/fiber cell gene or downregulated/epithelial cell gene) that also have an increase in average methylation (more methylated in fiber cells compared to epithelial cells) or decrease in average methylation (demethylated in fiber cells compared to epithelial cells) across the promoter and genebody combined. Data indicate only DEGs that contain at least one mCG DMR. *χ*^2^ test, *p* < 1 × 10^–43^. **C** Same as **A** but only the average change in methylation of mCG DMRs mapped to putative promoters only. Data indicate only DEGs with putative promoters that contain at least one mCG DMR. Pearson correlation *r* = − 0.39, *p* < 1 × 10^–11^. **D** Same as **B** but only the average change in methylation of mCG DMRs mapped to putative promoters only. Data indicate only DEGs with putative promoters that contain at least one mCG DMR. *χ*^2^ test, *p* < 1 × 10^–9^

To determine the relationship between mCG DNA methylation changes and gene expression changes occurring during the epithelial cell to fiber cell transition, we integrated the bisulfite sequencing methylation data with the RNA-seq data and quantified the average change in mCG methylation for all differentially methylated regions that mapped to the gene bodies and putative promoters (− 2 kb upstream from transcription start sites) of differentially expressed genes (log2FC > 0.4 or < − 0.4, and *q* < 0.05) (Additional file 9: Table S5A–D). Of all differentially expressed genes detected between epithelial cells and fiber cells (log2-fold change FPKM > 0.4 or < − 0.4 and *q* < 0.05), 1063 exhibited increased average mCG methylation levels in fiber cells, while 222 genes exhibited decreased average mCG methylation levels in fiber cells in gene bodies and putative promoter regions (up to – 2 kb upstream from the transcription start site). A plot of the log2-fold change in gene expression levels vs the average difference in mCG methylation levels reveals a significant negative correlation between methylation changes and gene expression changes of those genes identified to exhibit different expression levels between lens epithelial and fiber cells (Fig. 4A, Pearson correlation *r* = − 0.37, *p* < 1 × 10^–42^). These data suggest that increased mCG methylation of gene bodies and putative promoter regions plays a significant role in the regulation of genes during fiber cell differentiation. 814 of 1063 genes with increased mCG methylation levels (76.58%) in fiber cells also exhibited decreased expression levels in fiber cells relative to epithelial cells (Fig. 4B, Additional file 9: Table S5A). Consistently, 159 of 222 genes exhibiting decreased mCG methylation levels in fiber cells (71.62%) also exhibited increased expression levels in fiber cells compared to epithelial cells (Fig. 4B, Additional file 9: Table S5B), indicating a very significant association between the identified mCG methylation and gene expression changes (Chi-squared test, *p* < 1 × 10^–43^).

Enrichr analysis of the 814 epithelial cell genes with increased mCG methylation levels in fiber cells (Additional file 9: Table S5A) revealed significant associations with epithelial-to-mesenchymal transition, Notch signaling, UV response, cell cycle control, hypoxia, and regulation of transcription (Additional file 3: Figure S3A, B, Additional file 10: Table S6A, B). Of the 159 fiber cell genes with decreased mCG methylation in fibers (Additional file 9: Table S5B), Enrichr analysis revealed significant functions in hedgehog signaling, cell–cell junction organization, intermediate filament organization, cytoplasmic translation and mRNA catabolism (Additional file 3: Figure S3C, D, Additional file 10: Table S6C, D). Collectively, these data suggest that changes in DNA methylation play a significant role in the regulated expression of genes preferentially expressed in either epithelial or fiber cell populations and could indirectly regulate multiple cellular features and functions.

We also conducted a similar analysis restricted to genes with differentially mCG methylated regions within putative promoter regions (up to − 2 kb upstream from gene transcription start sites) (Additional file 9: Table S5E–H). Of all differentially expressed genes detected between epithelial cells and fiber cells (log2-fold change FPKM > 0.4 or < − 0.4 and *q* < 0.05), 266 exhibited increased average mCG methylation levels in putative promoter regions (− 2 kb upstream from transcription start site) in fiber cells, while 45 genes exhibited decreased average mCG methylation levels in putative promoter regions (− 2 kb upstream from transcription start site) in fiber cells. A plot of the log2-fold change in gene expression levels vs the average difference in putative promoter mCG methylation levels revealed a significant negative correlation between putative promoter methylation changes and gene expression changes of those genes identified to exhibit different expression levels between lens epithelial and fiber cells (Fig. 4C, Pearson correlation *r* = − 0.39, *p* < 1 × 10^–11^). These data suggest that increased mCG methylation of putative promoter regions plays a significant role in the negative regulation of genes during fiber cell differentiation. 208 of 266 genes (78.20%) with increased putative promoter mCG methylation levels in fiber cells also exhibited decreased expression levels in fiber cells relative to epithelial cells (Fig. 4D, Additional file 9: Table S5E). By contrast, 30 of 45 genes (66.67%) with decreased putative promoter mCG methylation levels in fiber cells (65.12%) also exhibited increased expression levels in fiber cells relative to epithelial cells (Fig. 4D, Additional file 9: Table S5F). A Chi-squared test also confirmed a significant association between the identified methylation and gene expression changes (*p* < 1 × 10^–9^).

Enrichr analysis of the 208 epithelial cell genes with increased putative promoter mCG methylation levels in fiber cells (Additional file 9: Table S5E) revealed significant associations with epithelial-to-mesenchymal transition, Notch signaling, cell cycle control, hypoxia, mTORC1 signaling, hedgehog signaling, and glycolysis (Additional file 3: Figure S3E, F, Additional file 10: Table S6E, F). Of the 30 fiber cell genes with decreased putative promoter mCG methylation in fibers (Additional file 9: Table S5F), Enrichr analysis revealed significant functions in IFN-gamma response, actin filament organization, cell–matrix adhesion, eye development, and negative regulation of epithelial cell proliferation (Additional file 3: Figure S3G, H, Additional file 10: Table S6G, H). Collectively, these data suggest that DNA methylation changes within promoter regions play a significant role in the regulation of differentiation state-specific gene expression during lens cell differentiation and hence in the modulation of cell-type specific functions ranging from structure to homeostasis.

In summary, this analysis provides evidence that DNA methylation of promoter regions, and gene bodies plays a significant role in regulating the differentiation state-specific expression of multiple genes important for a wide variety of lens epithelial cell or fiber cell functions.

### Differentiation state-specific methylation patterns parallel changes on chromatin accessibility occurring upon lens fiber cell differentiation

**Fig. 5:**
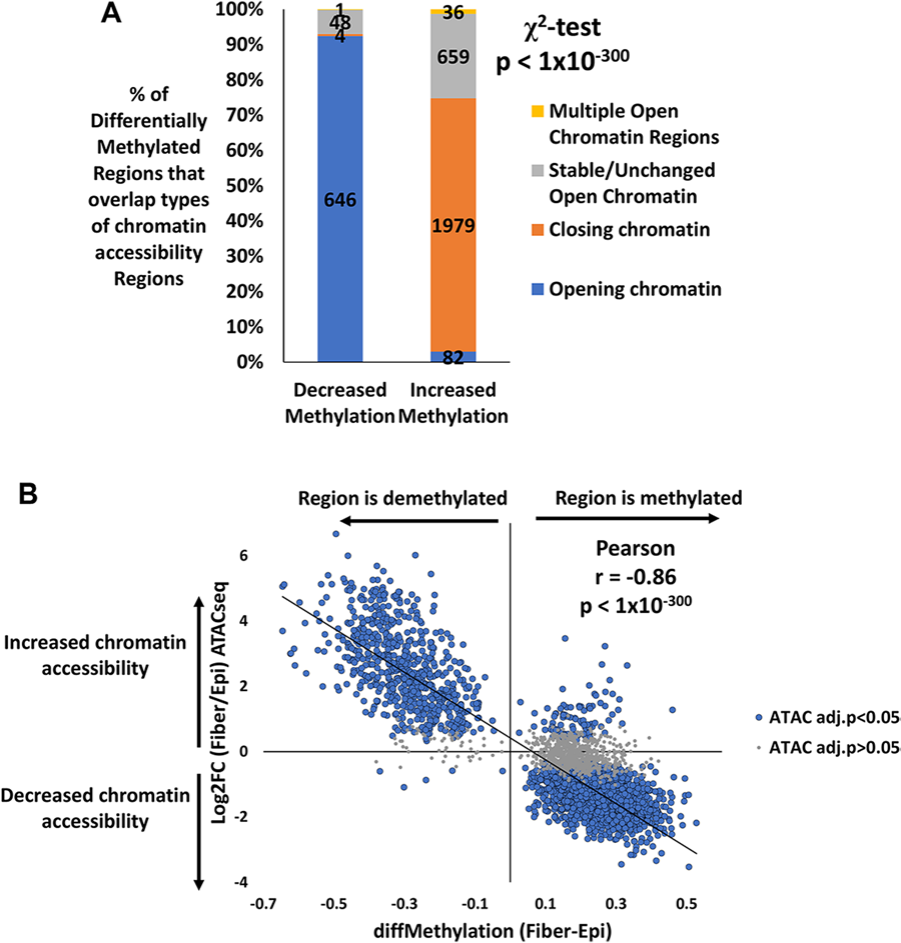
Differentiation-state-specific methylation patterns parallel changes on chromatin accessibility occurring upon lens fiber cell differentiation. **A** Proportion of mCG DMRs that overlap a chromatin-accessible region in the lens genome. Opening chromatin indicates a chromatin-accessible region that is significantly more open in lens fiber cells compared to lens epithelial cells (ATACseq log2FC > 0, adjusted *p* < 0.05). Closing chromatin indicates the inverse (ATACseq log2FC < 0, adjusted *p* < 0.05). Stable/unchanged open chromatin indicates a chromatin-accessible region that does not change significantly between lens fiber cells and lens epithelial cells (ATACseq adjusted *p* > 0.05). This plot is limited only to mCG DMRs that overlap at least one chromatin-accessible region. *χ*^2^ test, *p* < 1 × 10^–300^. **B** Scatter plot of the change in methylation (diff. methyl) of mCG DMRs plotted against the change in chromatin accessibility at the same region. The plot is divided into significant changes in chromatin accessibility (ATACseq adjusted *p* < 0.05) and non-significant changes (adjusted *p* > 0.05). This plot is limited only to mCG DMRs that overlap at least one chromatin-accessible region. Pearson correlation *r* = − 0.86, *p* < 1 × 10^–300^

To identify a possible relationship between DNA methylation and chromatin accessibility, we integrated bisulfite sequencing data and ATAC-seq data [32]. The analysis revealed that 699 regions of DNA exhibiting decreased levels of mCG DNA methylation in fiber cells and 2756 regions of DNA exhibiting increased levels of mCG DNA methylation in fiber cells were contained in chromatin-accessible regions in the lens genome of embryonic day 13 chick lenses. Analysis of this data revealed that 92% (646 of 699) of the regions exhibiting decreased levels of mCG DNA methylation in fiber cells were significantly associated with increased chromatin accessibility in fiber cells relative to epithelia (log2fc > 0.25 and *q* < 0.05) (Fig. 5A, Chi-square *p* < 1 × 10^–300^, Additional file 11: Table S7). Conversely, 72% (1979 of 2756) of the regions exhibiting increased levels of mCG DNA methylation in fiber cells was significantly associated with decreased chromatin accessibility in fiber cells relative to epithelia (log2fc < − 0.25 and *q* < 0.05). Consistently, a significant negative correlation was detected between changes in mCG methylation and chromatin accessibility (Fig. 5B, Pearson correlation *r* = − 0.86, *p* < 1 × 10^–300^), with regions showing the largest decreases in mCG methylation also showing the largest increases in accessibility and regions showing the largest increases in mCG methylation also showing the largest decreases in accessibility. Thus, there is a strong correlation between mCG DNA methylation state and chromatin accessibility in differentiating lens cells.

### Combined analysis reveals strong correlations between DNA methylation, chromatin accessibility and gene regulation during lens cell differentiation

**Fig. 6:**
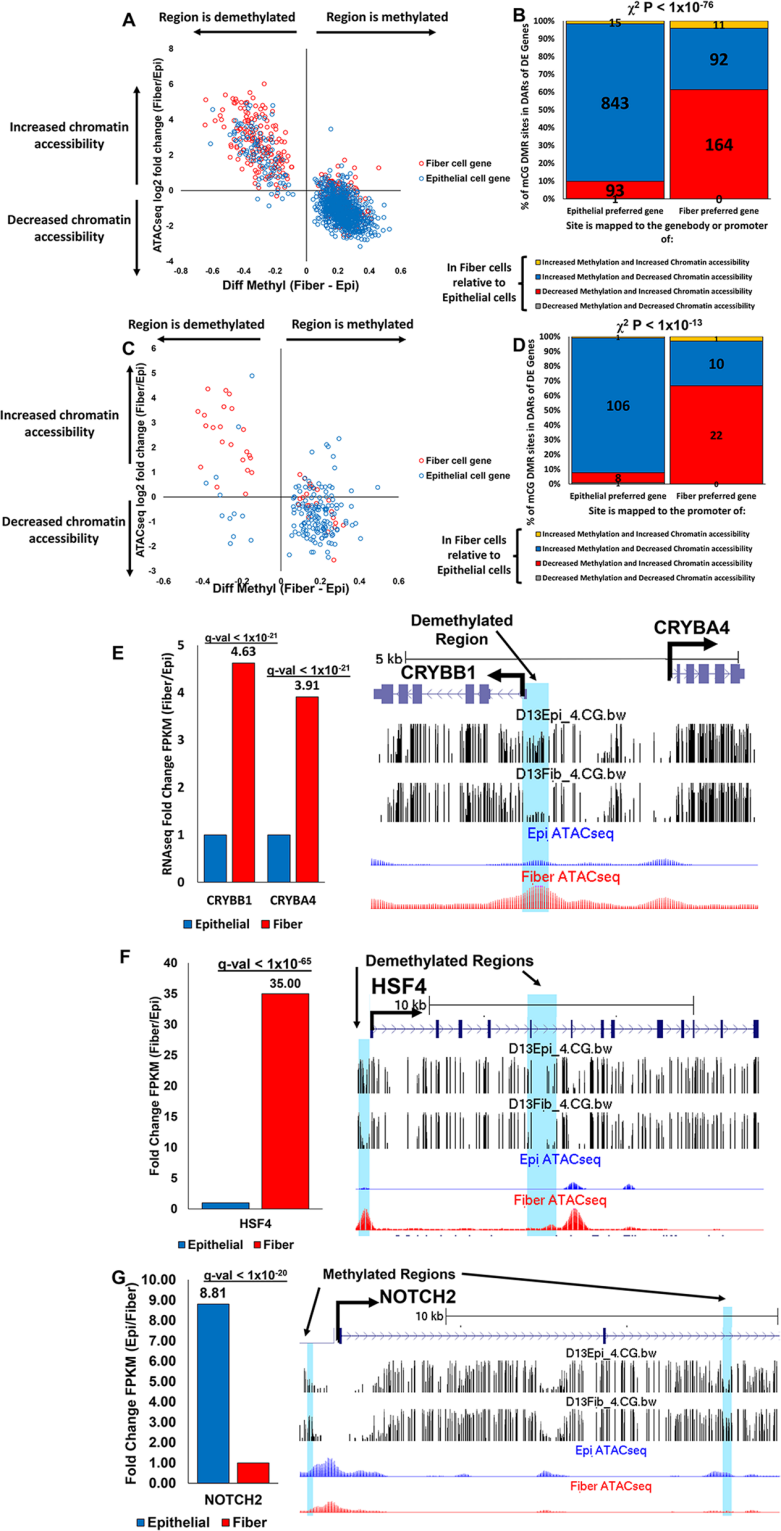
Multiomics analysis reveals strong correlations between DNA methylation, chromatin accessibility and gene regulation during lens cell differentiation. **A** Scatter plot of the change in methylation (diff. methyl) of mCG DMRs mapped to the promoter or genebody of a DEG plotted against the change in chromatin accessibility. Red indicates a mCG DMR that is mapped to an upregulated/fiber cell gene. Blue indicates a mCG DMR that is mapped to a downregulated/epithelial cell gene. This plot is limited only to mCG DMRs that are mapped both to a chromatin-accessible region and either the promoter or genebody of a DEG. **B** Proportion of differentially methylated regions contained within the promoter or gene body of a DEG (upregulated/fiber cell gene or downregulated/epithelial cell gene) that is also contained within a differentially accessible chromatin region. Each site is categorized based on whether it has (1) increased or decreased mCG methylation during lens differentiation; (2) increased or decreased chromatin accessibility during lens differentiation, and (3) if the site is in the promoter or gene body of an epithelial cell preferred gene (RNAseq log2FC < − 0.4, *q* < 0.05) or a fiber cell preferred gene (RNAseq log2FC > 0.4, *q* < 0.05). This chart is limited only to mCG DMRs that are mapped to a differentially accessible chromatin region (ATACseq *q* < 0.05) and either the promoter or genebody of a DEG. Chi-square test *p* < 1 × 10^–43^. **C** Same as **A** but limited to the change in methylation (diff. methyl) of mCG DMRs that are mapped only to the promoter of a DEG and a chromatin-accessible region. Red indicates a mCG DMR that is mapped to an upregulated/fiber cell gene. Blue indicates a mCG DMR that is mapped to a downregulated/epithelial cell gene. **D** Same as **B** but limited to the change in methylation (diff. methyl) of mCG DMRs that are mapped only to the promoter of a DEG that is also in a differentially accessible chromatin region (ATACseq *q* < 0.05). Chi-square test *p* < 1 × 10^–9^. **E** Galgal6 UCSC genome browser track encompassing CRYBB1 and CRYBA4. Black bent arrows indicate transcription start sites. Vertical light blue highlighted column indicates the genomic region within the promoter region of both CRYBB1 and CRYBA4 that contains a mCG DMR that is significantly demethylated in fiber cells compared to epithelial cells. Also shown are tracks of the relative mCG methylation levels in epithelial cells (D13Epi_4_CG.bw) and fiber cells (D13_Fib_4_CG.bw). Tracks of ATACseq peak intensity from lens epithelial and fiber cells. Taller and wider peaks represent increased chromatin accessibility at the corresponding genomic region. Also shown are the relative gene expression changes expressed as fold change FPKM values of CRYBB1 and CRYBA4 in fiber cells compared to epithelial cells. **F** Same as **C** but for HSF4. Two mCG DMRs are shown. One in the promoter region (left) and one in the genebody (right). The relative gene expression changes expressed as fold change FPKM values of HSF4 in fiber cells compared to epithelial cells. **G** Same as **C** but for NOTCH2. Two mCG DMRs are shown. One in the promoter region (left) and one in the genebody (right). The relative gene expression changes of NOTCH2 are expressed as fold change FPKM values in epithelial cells compared to fiber cells

Integration of bisulfite sequencing, ATACseq, and RNAseq data comparing lens epithelial cells and fiber cells during lens differentiation, revealed 1219 sites in gene bodies and promoters of differentiation state-specific genes that also have significant changes in DNA methylation and chromatin accessibility (Fig. 6A, B, Additional file 12: Table S8A). The combined analysis reveals that 164 sites within gene bodies or promoters of fiber cell genes exhibit decreased methylation and increased chromatin accessibility. One hundred and thirty-five fiber cell preferred genes contained at least one demethylated DNA region that also exhibited increased chromatin accessibility. Additionally, the analysis revealed that 843 identified sites within gene bodies or promoters of epithelial cell preferred genes exhibit increased methylation and decreased chromatin accessibility in fiber cells, and 467 epithelial cell preferred genes contain at least one methylated region that also exhibits decreased chromatin accessibility. A Chi-squared test confirms the significant association between differentially methylated mCG sites in differentially accessible chromatin regions and corresponding gene expression changes (Fig. 6B, Chi-square *p* < 1 × 10^–76^). A similar analysis was performed for the 149 sites in promoters of differentiation state-specific genes that also show significant changes in DNA methylation and chromatin accessibility (Fig. 6C, D, Additional file 12: Table S8B). Similar to the results above, 22 sites within promoters of fiber cell preferred genes exhibit decreased methylation and increased chromatin accessibility. 22 fiber cell preferred genes contained at least one demethylated DNA region that also exhibited increased chromatin accessibility. Additionally, the analysis revealed that 106 identified sites within promoters of epithelial cell preferred genes exhibited increased methylation and decreased chromatin accessibility in fiber cells. 91 epithelial cell preferred genes contained at least one methylated region that also exhibited decreased chromatin accessibility in fiber cells. A Chi-squared test confirmed a significant association between differentially methylated mCG sites in differentially accessible chromatin regions at putative promoters (− 2 kb upstream from the transcription start site) and corresponding gene expression changes (Fig. 6D, Chi-square *p* < 1 × 10^–13^). These data suggest a direct relationship between DNA methylation and chromatin accessibility both of which are correlated with specific changes in gene expression occurring during the differentiation of lens epithelial cells into lens fiber cells.

This relationship extends to multiple genes with important lens functions. Both of the crystallin genes CRYBB1 and CRYBA4 [55–58] share the same promoter region, a feature also seen in mouse and human genomes [59, 60], and both have at least 3 × greater expression levels in fiber cells compared to epithelial cells (Fig. 6E). The shared promoter region contains one differentially methylated region with significantly lower methylation levels in fiber cells compared to epithelial cells. This demethylated region is also contained within an open chromatin region with increased chromatin accessibility in fiber cells compared to epithelial cells. Another example is the lens fiber cell-specific transcription factor HSF4 [61, 62] (Fig. 6F) that exhibits one differentially methylated region in the promoter and one in the fifth intron of the gene body. Both have significantly lower methylation levels, and both are also contained within an open chromatin region with increased chromatin accessibility in fiber cells compared to epithelial cells. There is also an open chromatin region partially contained within the sixth intron and sixth exon that exhibits increased chromatin accessibility in fiber cells compared to epithelial cells and also appears to exhibit lower methylation levels in fiber cells relative to epithelial cells. However, this region was not identified as a statistically significant differentially methylated region by the DSS analysis software. Another example is NOTCH2, a receptor that upon conditional mutation results in a cataract in mice [63]. The Notch signaling pathway has been previously established as an essential pathway for lens differentiation [64]. NOTCH2 (Fig. 6G) is expressed at significantly lower levels in fiber cells and contains one differentially methylated region in the promoter and one in the second intron of the gene body. Both regions have significantly higher methylation levels in fiber cells. The methylated region in the promoter is contained within an open chromatin region with unchanged chromatin accessibility (ATACseq *q* > 0.05) and the methylated region in the second intron is contained within an open chromatin region with decreased chromatin accessibility in fiber cells (ATACseq log2FC < 0, *q* < 0.05). The genome tracks used for Fig. 6 can also be found at (http://genome.ucsc.edu/s/jdisatha/galGal6_methylome_Lens) where interested readers can also search for any gene of interest to visualize the presence or absence of nearby differentially methylated regions and differential chromatin accessibility regions between lens epithelial and fiber cells.

### Identification of transcription factor binding sites in differentially methylated DNA regions

**Fig. 7:**
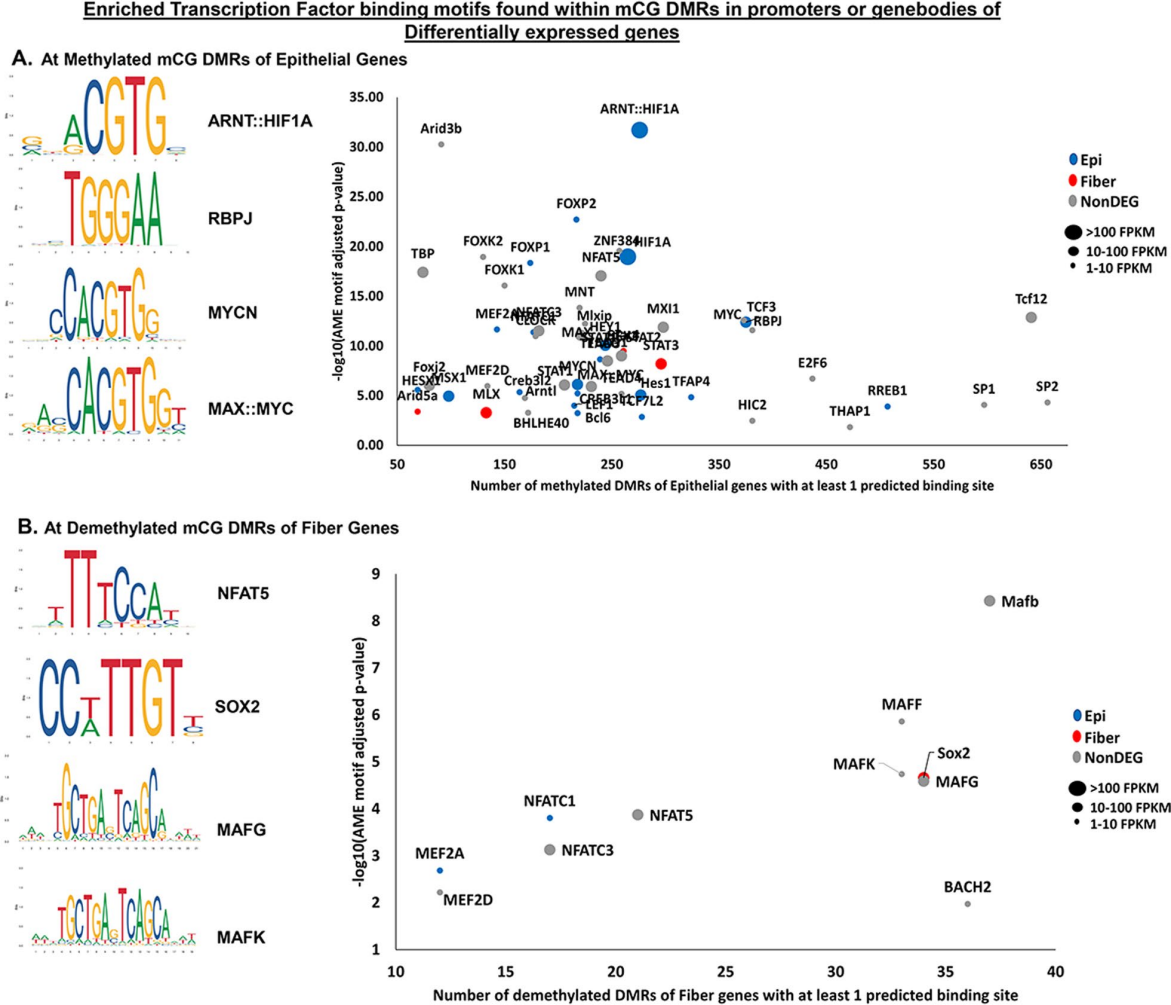
Identification of transcription factor binding sites in differentially methylated DNA regions. **A** DNA sequences within Genomic regions with (1) significantly higher methylation in lens fiber cells compared to lens epithelial cells and (2) found in the genebodies or promoters of genes with decreased expression levels in fiber cells compared to epithelial cells were analyzed to find significantly enriched transcription factor binding motifs. Scatterplot shows a plot of the − log10 (adjusted *p* value) of significantly detected transcription factor motifs identified using the AME tool versus the number of downregulated/epithelial cell genes that have at least one binding site within a methylated region identified by the FIMO tool. Only transcription factors encoded by genes that have a total transcript expression level (sum of average Epi and Fiber FPKM) of at least 1 FPKM are reported (Additional file 13: Table S9A). The color of each datapoint also indicates whether the gene encoding the transcription factor is more highly expressed in epithelial cells (blue) (log2-fold change FPKM < − 0.4, *q* < 0.05), fiber cells (red) (log2-fold change FPKM > 0.4, *q* < 0.05), or there is no significant differences in gene expression levels between epithelial and fiber cells (grey) (Additional file 13: Table S9A). The size of each datapoint corresponds to the total transcript expression level for each transcription factor. Select transcription factor consensus sequence logos from JASPAR database are shown. **B** Same as **A** but for DNA sequences in genomic regions with (1) significantly lower methylation in lens fiber cells compared to lens epithelial cells and (2) found in the genebodies or promoters of genes with increased expression levels in fiber cells compared to epithelial cells

To explore the relationship between representation of transcription factor consensus sequences in regions with altered DNA methylation and altered gene expression, transcription factor binding motifs significantly overrepresented in differentially methylated DNA regions were correlated with differential gene expression levels in lens epithelial and fiber cells using the AME tool from MEME-suite [65, 66] (Fig. 7A, B). Regions of DNA with significantly greater methylation in fiber cells relative to epithelial cells and mapped to promoters or gene bodies of epithelial cell genes contained, among others, the binding motifs of ARNT:HIF1a, RBPJ, MYCN, and MAX:MYC (Fig. 7A, Additional file 13: Table S9A). Many of the enriched binding motifs are from transcription factors with well-established roles in regulation of lens gene expression, lens development, and differentiation (Additional file 13: Table S9) [44, 64, 67–89]. Consistently, previous studies showed HIF1 to be a major regulator of lens gene expression [44], RBPJ is involved in early lens development [64, 87], and both c-Myc and n-Myc have established roles in lens cell proliferation and differentiation [70, 86]. Conversely, regions of DNA that exhibited decreased methylation in fiber cells and are contained within putative promoters of genes preferentially expressed in fiber cells contained, among other binding sequences, the consensus site for transcription factor NFAT5, SOX2, and the MAF family of transcription factors (Fig. 7B, Additional file 13: Table S9B). Consistently, previous studies revealed a role for NFAT5 in the hyperosmotic stress response in human lens epithelial cells [85], SOX2 is a well-established transcription factor required for lens development [90], and the MAF family of transcription factors are known regulators of lens gene expression and lens development [76, 77, 79–81]. The data suggest that changes in DNA methylation within the identified regions regulate the ability of specific lens transcription factors to control gene expression changes during lens cell differentiation.

Transcription factor abundance is one of many factors that control the rate of transcription of target genes [91]. Although the present study does not reveal the relative protein abundances of transcription factors, the analysis does identify the relative mRNA expression levels of the transcription factors with the most enriched consensus sequences found within differentially methylated regions of DNA. Many of the highly expressed transcription factors identified in Fig. 7 and Additional file 13: Table S9 (total FPKM > 12) such as HIF1A [44, 92], HEY1 [88], STAT3 [69], SOX2 [82], and MAFB [76, 77, 79, 80] have known functions in lens development and differentiation (Additional file 13: Table S9). In contrast, the transcription factors with low expression levels (total FPKM < 5) such as MAFF, Arid5a, TEAD4, and HIC2 (Additional file 13: Table S9) do not yet have any known functions in the regulation of lens structure, homeostasis, or gene expression. Although we cannot rule out the potential role of lowly expressed transcription factors in the regulation of the identified differentially methylated genes, the data suggest that transcription factors that are (1) highly expressed, and (2) have a highly enriched consensus sequence in the identified differentially methylated regions (high − log10 AME motif adjusted *p* value and high number of predicted target genes, Fig. 7, Additional file 13: Table S9) likely play a major role in the regulation of lens gene expression via binding to differentially methylated regulatory elements during lens differentiation.

Interestingly, the SOX2 gene is more highly expressed and demethylated in fiber cells relative to epithelial cells (Additional file 13: Table S9B). Analysis of transcription factor binding sequences revealed that the consensus sequence for SOX2 is enriched in genes that are more highly expressed and demethylated in fiber cells relative to epithelial cells. Thus, both SOX2, and its predicted target fiber cell genes (Additional file 14: Table S10A) have increased expression and decreased methylation during lens cell differentiation. Conversely, the genes for RREB1, TCF3, FOXP1, CREB3L2, HIF1a, BCL6, MEF2A, MSX1, LEF1, CREB3L1, HEY1, TCF7L2, and NFATC1 have significantly decreased expression levels and are methylated in fiber cells relative to epithelial cells (Additional file 13: Table S9A). Analysis of transcription factor binding sequences revealed that the consensus sequences for these transcription factors are enriched in genes with decreased expression and increased methylation in fiber cells relative to epithelial cells. Thus, both the identified transcription factors and their predicted target epithelial cell genes (Additional file 14: Table S10B) have decreased expression and increased methylation during lens cell differentiation. The data suggest a role for DNA methylation in controlling both the availability and accessibility of transcription factors during lens differentiation.

## Discussion

Although the role of CG methylation (mCG) in gene silencing is well-established, few studies have examined its potential role in control of cellular differentiation. In the present study, we sought to establish a role for CG methylation in control of gene expression levels required for differentiation of immature lens epithelial cells into mature fiber cells conferring the adult structure and transparent function of the eye lens. The lens is a well-characterized model for cellular differentiation and previous studies have shown that differentiation state-specific changes in gene expression [43, 93] and chromatin accessibility [32, 47] characterize the differentiation of lens epithelial cells into lens fiber cells. Here, we employed bisulfite sequencing to interrogate the potential for mCG methylation in regulating chromatin accessibility, gene expression, and hence lens cell differentiation.

Analysis of whole genome bisulfite sequencing data identified 7621 genomic regions exhibiting differentiation state-specific differences in mCG levels between undifferentiated lens epithelial and differentiated fiber cells. Sites exhibiting increased mCG methylation at promoters and genebodies had a strong correlation with decreased gene expression levels in lens fiber cells and sites exhibiting decreased mCG methylation at promoters and genebodies had a strong correlation with increased gene expression levels in lens fiber cells (Pearson correlation *r* = − 0.37, *p* < 1 × 10^–42^).

Interrogation of the relationship between mCG DNA methylation levels and chromatin accessibility changes determined by ATAC-seq revealed that sites exhibiting decreased mCG methylation had a strong correlation with increased chromatin accessibility in lens fiber cells and sites exhibiting increased mCG methylation had a strong correlation with decreased chromatin accessibility in lens fiber cells (Pearson correlation *r* = − 0.86, *p* < 1 × 10^–300^).

Many of the identified genes exhibiting altered mCG DNA methylation changes in association with corresponding changes in chromatin accessibility and differentiation state-specific gene expression have established requirements for specialized lens functions including lens cell structure, homeostasis and cell signaling (Additional file 15: Table S11) [41, 44, 56, 62–64, 75, 82, 92, 94–119, 119–184]. For instance, the lens crystallins, CRYBB2, CRYBB1, CRYBA4, CRYGN, CRYBB3, and CRYBA1 are all critical for lens fiber cell transparency. With the exception of CRYBB2, all these genes are more highly expressed in fiber cells. CRYBA1, CRYBB2, and CRYBB3 are more highly methylated in fiber cells while conversely CRYBA4, CRYBB1, and CRYGN are demethylated in fiber cells, showing the complexity of this level of control. However, the genes in each group are coordinately expressed through epithelial to fiber cell differentiation (Ma et al., submitted*)*. δ-crystallin (ASL1) is the most highly expressed crystallin in chicken [185, 186] and is more highly expressed in fiber cells compared to epithelial cells (Additional file 7: Table S3). However, there were no detectable changes in mCG DNA methylation in the promoter nor genebodies of these genes. This suggests that the differential expression of δ-crystallin is likely controlled by a mechanism independent of DNA methylation changes. Analysis of the ATACseq data from [32] reveals that the genebody and promoter of the δ-crystallin gene (ASL1) contains 6 regions of chromatin with increased accessibility in fiber cells compared to epithelial cells. Thus, it is likely that δ-crystallin expression is regulated by chromatin accessibility changes independent of DNA methylation changes.

Other non-crystallin genes important for lens cell homeostasis, structure, and transparency also exhibited altered mCG DNA methylation changes in association with corresponding changes in chromatin accessibility and differentiation state-specific gene expression. An example is HSF4 that is a fiber cell-specific transcription factor that regulates nuclear degradation during lens fiber cell differentiation [95, 187, 188]. Also included are the beaded filament proteins BFSP1 and BFSP2 that are well-established lens fiber cell structural genes [177]. NOTCH1, NOTCH2, HES5, and HEY1 are all components of the NOTCH signaling pathway which plays a major role in lens epithelial cell function and differentiation into fiber cells [63, 64, 189, 190]. Finally, the hypoxic environment of the lens itself has been shown to be critical for initiation of lens differentiation [31]. Consistent with a role for DNA methylation in regulating hypoxia-associated gene expression and lens properties, VEGF, a hypoxia-regulated lens gene involved in lens development [44, 191] and the hypoxia-regulated transcription factor HIF1a that controls lens fiber cell gene expression and the degradation of non-nuclear organelles that occurs during mature lens formation [44, 67] were identified as differentially methylated during lens cell differentiation.

A majority of lens-related function genes (89 of 112 genes, 79.5%) (Additional file 15: Table S11) had expression level changes associated with the inverse change in methylation levels consistent with the analysis performed on the entire set of 1285 differentially expressed genes with changes in methylation levels (973 of 1285 genes, 75.7% Fig. 4, Additional file 9: Table S5). Additionally, although a majority of the identified differentially expressed genes with differentially methylated regions do not yet have a reported function in the lens (1173 of 1285 genes, 91.3%), it is likely that these genes have some function in the lens but to-date these potential functions are not yet well-characterized or reported in the literature.

In addition to individual genes, many of the identified processes and pathways exhibiting an association between gene expression, and DNA methylation (Additional file 3: Figure S3, Additional file 10: Table S6) have well-established functions in the lens such as hedgehog signaling [192, 193], EMT [194, 195], UV response [196, 197], Notch signaling [63, 64, 139], cell cycle control [119, 198–200], hypoxia [44, 92], IFN-gamma signaling [201, 202]. The present data suggest that this epigenetic regulatory mechanism plays a role in these critical lens processes through changes in differentiation state-specific DNA methylation at potential cis-regulatory sequences.

Analysis of transcription factor binding sequences contained within DNA regions exhibiting differentiation state-specific DNA methylation changes revealed a wide variety of established and novel enriched transcription factor binding sequences (Additional file 13: Table S9) including those for HIF1a that has been previously established to be an important regulator of differentiation state-specific gene expression in the lens [44, 67], SOX2 which has a well-established role in lens development [82, 203, 204], and TCF3 that has yet to be evaluated for its potential lens function. Intriguingly, several of the genes encoding the identified transcription factors with enriched consensus sequences also had significant changes in methylation levels and gene expression levels in fiber cells compared to epithelial cells (Additional file 13: Table S9). Additionally, analysis of transcription factor binding sequences also revealed other enriched binding sequences for transcription factors that have previously established lens functions (Additional file 13: Table S9). These include the MAF family of transcription factors MAFB, MAFK, and MAFG which are known to regulate lens gene expression during development and differentiation [76, 77, 79–81], n-myc and c-myc [70, 86], and the NOTCH transcription factors RBPJ, and HES1 [64, 71, 87]. Some of the identified transcription factors may be unable to bind to chromatin due to DNA methylation reducing the accessibility to that site. This mechanism has been suggested in other studies [8, 14–18]. Therefore, it is likely that the transcription factors identified in Fig. 7A and Additional file 13: Table S9A are important for the expression of genes in epithelial cells. Then during differentiation and the formation of lens fiber cells, the transcription factor binding sites likely become obstructed due to increased mCG DNA methylation thus inhibiting the ability of these transcription factors to induce epithelial cell genes in the fiber cells. In contrast, it is likely that the transcription factors identified in Fig. 7B and Additional file 13: Table S9B are important for the increased expression of genes in fiber cells. During differentiation, these transcription factor binding sites are more accessible due to decreased mCG DNA methylation thus allowing the identified transcription factors to induce expression of genes in the fiber cells. The data suggest a role for DNA methylation in controlling the accessibility of transcription factors to predicted regulatory elements to control gene expression changes during lens differentiation. Additionally, many of the genes encoding the identified transcription factors have significant changes in methylation in their respective promoters and genebodies that are associated with significant changes in expression levels of these transcription factors during lens differentiation. The data suggest that DNA methylation may also control the availability of these transcription factors by regulating the expression of these transcription factors.

MYC (c-myc) a transcription factor that regulates lens cell proliferation during lens development [86] is one transcription factor predicted to bind to regions of DNA with increased mCG DNA methylation of genes with decreased expression levels in lens fiber cells compared to epithelial cells (Fig. 7A, Additional file 13: Table S9A). A previous study has shown that MYC interacts with the DNA methyltransferase DNMT3A to directly methylate CG dinucleotides of the CDKN1A, CCND1, and TIMP2 genes in U373 cells [205] leading to decreased expression levels of these genes. Analysis of the present bisulfite sequencing data revealed that CCND1 and TIMP2 contain multiple regions of DNA with increased mCG DNA methylation in lens fiber cells compared to epithelial cells (Additional file 6: Table S2). Consistently, CCND1 is a Wnt signaling gene in lens epithelial cells that is known to regulate the cell cycle and promote lens epithelial cell proliferation [165]. Its expression levels decrease in fiber cells in parallel with cell cycle exit and terminal lens fiber cell differentiation (Additional file 7: Table S3A, Additional file 15: Table S11)[119]. Analysis of the DNA sequences contained within the regions of CCND1 that have increased mCG DNA methylation, revealed 3 sites that had the MYC transcription factor consensus sequence (Additional file 14: Table S10B). These data suggest that the transcription factor MYC (c-myc) could be directing DNMT3A to methylate CCND1 to inhibit its expression, thus promoting cell-cycle exit leading to terminal lens fiber cell differentiation. Further experiments are required to validate the potential regulatory effect of MYC-DNMT3A on CCND1 expression in the lens and to fully elucidate the potential effects on lens epithelial cell-cycle exit followed by lens fiber cell differentiation.

Intriguingly, despite the overall trend of increased global DNA methylation in differentiated lens fiber cells relative to undifferentiated lens epithelial cells, a large number of genes more highly expressed in lens fiber cells exhibited demethylation relative to epithelial cells. These results suggest that regulation of gene expression by methylation and demethylation could both negatively and positively regulate the expression of genes specific for lens epithelial or fiber cells and they implicate differentiation state-specific control by DNA methylases in this coordinate regulation. An examination of expression levels of methylases between undifferentiated epithelial cells and differentiated fiber cells presents DNMT1 and DNMT3A as non-differentially expressed, while DNMT3B is more highly expressed in epithelial cells relative to fiber cells (Additional file 7: Table S3). Additionally, the DNA demethylases TET2, and TET3 are not differentially expressed, while TET1 is more highly expressed in epithelial cells relative to fiber cells (Additional file 7: Table S3). Of these DNA methylation genes, the DNMT3A genebody and TET2 promoter have increased mCG DNA methylation while the TET1 promoter has decreased mCG DNA methylation in fiber cells relative to epithelial cells (Additional file 6: Table S2). Consistently, lens-specific deletion of DNMT1 results in apoptosis of lens epithelial cells, abnormal lens fiber cell morphology, and specific changes in lens gene expression [206]. Correspondingly, HIF1a which was detected to be methylated and silenced during lens cell differentiation in the present study exhibited increased expression upon DMNT1 deletion in the lens [206] suggesting a role for DNMT1 in controlling HIF1a expression levels. Consistent with DNMT1 playing a general role in the regulation of cell differentiation, overexpression of DNMT1 in breast cancer, pituitary adenomas, and B-cell lymphoma resulted in alteration of both CG methylation and gene expression [207–209].

The integration of bisulfite sequencing and RNAseq data revealed an inverse relationship between DNA methylation levels and gene expression during lens cell differentiation. This negative correlation has also been observed in other important studies in multiple species and other tissues including human trophoblast differentiation [23], myogenic differentiation [21], umbilical cord blood derived mononuclear cells to endothelial cell differentiation [210], adipogenesis [22], and chondrocyte hypertrophic differentiation [24]. Of the 1285 differentially expressed genes that contained at least 1 differentially methylated region within the genebody or promoter (Additional file 9: Table S5A–D), a majority (974 genes, 75.8%) contained differentially methylated regions only within the genebody, while 154 genes (12.0%) contained differentially methylated regions only within the promoter, and 157 genes (12.2%) contained differentially methylated regions in both the promoter and genebody. A Pearson correlation analysis comparing the DNA methylation levels and gene expression changes for these subsets of genes revealed that genes with differentially methylated regions in both the promoter and genebody are more inversely correlated with gene expression (Pearson *r* = − 0.49) compared to genes with differentially methylated regions only in the promoter (Pearson *r* = − 0.43), or only in the genebody (Pearson *r* = − 0.35). The data reveal a small variation that suggests methylation changes in the promoter may be more closely correlated with gene expression changes compared to methylation changes in the genebody. However, genes with methylation changes in both the genebody and promoter are even more closely correlated with gene expression changes.

The integration of bisulfite sequencing and ATACseq data also revealed an inverse relationship between DNA methylation levels and chromatin accessibility at differentially methylated regions during lens cell differentiation. This negative correlation between DNA methylation and chromatin accessibility has also been observed in multiple other studies in a variety of species and tissues [211–213]. Of the 3455 differentially methylated regions that are contained within open chromatin regions (Additional file 11: Table S7), a majority (2690, 77.9%) are within genebodies and a minority (237, 6.9%) are within putative promoters (− 2 kb from TSS). The remaining 528 differentially methylated regions in open chromatin are either mapped to intergenic regions or overlap both the promoter and genebody. Pearson correlation analysis revealed that the methylation changes at the 2690 differentially methylated regions within open chromatin at genebodies are more negatively correlated with changes in chromatin accessibility (Pearson *r* = − 0.87) compared to methylation changes at the 237 differentially methylated regions within open chromatin at putative promoters (Pearson *r* = − 0.80). The data reveal a small variation that suggests that methylation changes in the genebody are more closely correlated to chromatin accessibility changes compared to methylation changes in the promoter.

Interestingly, integration of the bisulfite sequencing, ATACseq, and RNAseq data revealed 135 genes preferentially expressed in fiber cells that also exhibited at least one genomic site with decreased mCG DNA methylation and increased chromatin accessibility. Conversely, there were 467 genes preferentially expressed in epithelial cells that also exhibited at least one genomic site with increased mCG DNA methylation and decreased chromatin accessibility. While they represent a minority of all differentially expressed genes (602 of 4582, 13.14%), within this group there is a strong association between epigenetic regulation of differentiation state-specific gene expression via DNA methylation and chromatin accessibility changes during lens cell differentiation. Further, although a majority of differentially expressed genes with differentially methylated regions exhibited an inverse relationship between mCG DNA methylation levels and gene expression changes (973 genes, Fig. 4B), a minority of genes (312) exhibited a positive correlation. This has also been seen in other studies [214, 215] and it is well known that the DNA methylation and chromatin accessibility landscapes do not always follow the inverse correlation with gene expression, implying that methylation is only one of multiples levels of transcriptional control [216]. Finally, only 1285 of the 4582 differentiation state-specific genes exhibited significantly altered mCG DNA methylation levels in the promoter or gene body. This observation suggests that a majority of gene expression changes during lens cell differentiation are regulated by alternative mechanisms independent of direct DNA methylation changes at potential regulatory regions. An analysis of the ATACseq data obtained from [217] revealed that 2178 of the 3297 (66.1%) differentiation state-specific genes without changes in mCG methylation contained at least one region of altered chromatin accessibility between lens epithelial and fiber cells. This suggests that chromatin accessibility changes are one likely regulatory mechanism controlling the expression of those genes that did not display mCG DNA methylation changes between lens epithelial and fiber cells.

The integration of bisulfite sequencing data, RNAseq data, and ATACseq data revealed that 717 differentially expressed genes had at least 1 differentially methylated region contained within a region of altered chromatin accessibility between lens epithelial and fiber cells (ATACseq *q* < 0.05, Additional file 12: Table S8). This suggests that both mCG DNA methylation changes and chromatin accessibility changes regulate the expression of these differentiation state-specific genes. However, it cannot be discerned from the present data whether DNA methylation changes are primary or secondary to chromatin accessibility changes regarding regulation of gene expression during lens differentiation. It is possible that DNA methylation changes could precede and regulate chromatin accessibility changes [218]. Or changes in chromatin accessibility could alter accessibility of DNA methyltransferases to target sites leading to altered DNA methylation patterns [216, 219]. Further experiments are required to fully elucidate the dynamic epigenetic landscape of the lens, the potential causal relationship between DNA methylation changes and chromatin accessibility changes, and subsequent control of gene expression during lens differentiation.

Although many differentially methylated mCG regions were located within open chromatin regions (3455 of 7621 mCG DMRs), only a small percentage of open chromatin regions contain differentially methylated regions (Additional file 4: Figure S4). This suggests that most chromatin accessibility changes likely occur independently of DNA methylation changes, or perhaps that the methylation background of these genes is set for the eye developmental field before D13 rather than specifically for the epithelial to fiber cell differentiation. Nevertheless, for those genes that are differentially methylated, changes in the level of methylation are a strong predictor of changes in chromatin accessibility and changes in gene expression levels.

A Pearson correlation analysis comparing gene expression changes and methylation changes within open chromatin regions (Additional file 16: Table S12) revealed that mCG methylation changes within regions of chromatin that have significant changes in chromatin accessibility (ATACseq adjusted *p* value < 0.05) are most negatively correlated with gene expression (*r* = − 0.49) compared to mCG methylation changes within any open chromatin region (*r* = − 0.44) and methylation changes at all DNA regions within the promoter and genebody (*r* = − 0.37, Fig. 4A). Additionally, limiting the analysis to only promoter mCG methylation changes within regions of chromatin with significant changes in chromatin accessibility revealed the most negative correlation with gene expression (*r* = − 0.57). Although they make up a small proportion of all differentially expressed genes (133 of 4582, 2.9%), they nevertheless represent the set of genes that are likely regulated by a combination of DNA methylation changes and chromatin accessibility changes, especially within putative promoters.

## Conclusions

Collectively, these results establish DNA methylation as a mechanism regulating the genes and processes associated with lens cell differentiation and implicate it in alterations in chromatin accessibility and hence the control of gene expression that characterize lens cell differentiation. They also suggest a role for DNA methylation in controlling both the availability and accessibility of transcription factors during lens differentiation and provide a basis for future studies on the role of epigenetic mechanisms in control of lens cell differentiation. Finally, they provide a general blueprint for studies aimed at identifying the role of DNA methylation in the development and differentiation of more complex tissues.

## Methods

### Microdissection and isolation of embryonic chick lens epithelial and fiber cells

Lenses were isolated from White Leghorn embryonated chicken eggs (Charles River Laboratories, Storrs, CT) at embryonic developmental stage day 13 (E13). 25 lenses per biological replicate were microdissected into epithelial cells and fiber cells as previously described [92]. Microdissected epithelial and fiber cells from 25 lenses were pooled and stored in CryoStor CS10 cryopreservation media containing 10% DMSO (Stemcell, Vancouver, Canada) and stored at − 80 °C as previously described [220].

### DNA isolation and whole genome bisulfite sequencing

Tissue in CryoStor media was thawed on ice, centrifuged at 800×*g* for 5 min and the CryStor removed. DNA was isolated using Qiagen Genomic-tip 20/G along with the Qiagen Genomic DNA buffer set according to the manufacturer’s instructions. Bisulfite sequencing was performed by Novogene (Sacramento CA) using an Illumina HiSeqTM2500/MiSeq platform using their standard protocols followed by CASAVA base calling and Trimmomatic read trimming and alignment to the galgal6 reference genome using Bismark software [221].

### Identification of methylated cytosines and differentially methylated regions

Reads mapped to chromosomes 1–30, Z, and W were retained. Fifteen–eighteen million methylated cytosines were identified across biological replicates of pooled lens epithelial and fiber cells. Fourteen–sixteen million methylated cytosines were of the mCG type. DSS analysis software based on beta-binomial distribution was used for the identification of DMRs as described previously [222, 223] with consideration of the spatial correlation, read depth of the sites, and the variance among biological replicates. The parameters were smoothing = TRUE, smoothing.span = 200, delta = 0, p.threshold = 1 × 10 − 5, minlen = 50, minCG = 3, dis.merge = 100, and pct.sig = 0.5. 7,621 differentially methylated mCG regions were identified between lens epithelial and fiber cells (Additional file 6: Table S2).

### RNA isolation, RNA-sequencing, and identification of differentially expressed genes

Total RNA was isolated from biological triplicates of microdissected chick lens epithelial and fiber cells using TRIZOL^®^ reagent (Invitrogen, prod no: 15593018) according to the manufacturer’s instructions. Libraries were prepared following Illumina’s TruSeq-stranded-total-RNA-sample preparation protocol. Paired-end sequencing was performed on Illumina’s NovaSeq 6000 platform. Cutadapt [224] was used to remove adaptor-contaminated reads, low quality bases and undetermined bases. Reads were mapped to galgal6 genome (ensemble version 96) using Bowtie2 [225] and HISAT2 [226]. The mapped reads were assembled using StringTie [227]. Comprehensive transcriptomes were generated using gffcompare and StringTie. The R package edgeR [228] was used to estimate the expression levels of all transcripts and to identify differentially expressed genes (log2-fold change FPKM > 0.4 or <  − 0.4) with parametric *F* test comparing nested linear models (*q* < 0.05). 4,582 genes were found to be differentially expressed between lens epithelial cells relative to lens fiber cells (Additional file 7: Table S3).

### Integrated analysis of bisulfite sequencing and RNA-sequencing data

Differentially methylated mCG regions were categorized based on the genomic loci the region was mapped to including promoter (− 2 kb upstream from transcription start site), utr5, exon, intron, utr3, CGI, CGI shore, or repeat region. Differentially methylated mCG regions at promoters or gene bodies (utr5, exon, intron, utr3) were compared to the expression pattern of the nearest gene. Since many genes contained more than one differentially methylated mCG region in the promoter and gene body, the average change in mCG methylation level for all mCG regions mapped to the promoter and genebody of each gene was calculated (Additional file 9: Table S5). Pearson correlation analysis and Chi-square analysis was conducted to determine the potential correlation and association between average changes in mCG methylation levels and changes in gene expression during lens differentiation from undifferentiated lens epithelial cells to differentiated lens fiber cells (Fig. 4A–D).

### Integrated analysis of bisulfite sequencing and ATAC sequencing data

Genomic coordinates of chick lens open chromatin regions mapped by ATAC sequencing [43] were lifted to galgal6 using UCSC Genome Browser [229] liftover tool. Open chromatin regions were categorized as opening (log2-fold change Fiber/Epi > 0, *q* < 0.05), closing (log2-fold change Fiber/Epi < 0, *q* < 0.05) or stable/unchanged (*q* > 0.05) based on data obtained from a previous study of ATAC sequencing on microdissected embryonic chick lens cells at the same developmental stage E13 [217].

Differentially methylated mCG regions identified by whole genome bisulfite sequencing that overlap the genomic coordinates of previously identified open chromatin regions by at least 1 bp are noted in Additional file 11: Table S7. 3455 differentially methylated mCG regions were found in open chromatin regions. Pearson correlation analysis and Chi-square analysis was conducted to determine the potential correlation and association between changes in mCG methylation levels and changes in chromatin accessibility during lens differentiation from undifferentiated lens epithelial cells to differentiated lens fiber cells (Fig. 5A, B).

### Approach to integrating bisulfite sequencing, RNA-sequencing, and ATAC sequencing

Differentially methylated mCG regions identified by whole genome bisulfite sequencing were filtered for both of the following properties: (1) region overlaps an open chromatin region with differentiation state-specific chromatin accessibility (log2-fold change Fiber/Epi ≠ 0, and *q* < 0.05) by at least 1 bp and (2) region is contained within the promoter (− 2 kb upstream of transcription start site) or gene body of a differentially expressed gene (FPKM log2FC > 0.4 or < − 0.4, and *q* < 0.05). The identified differentially methylated mCG regions were separated based on whether they mapped to the promoter or gene body of an epithelial cell preferred gene (RNAseq FPKM log2-fold change < − 0.4, and *q* < 0.05) or a fiber cell preferred gene (RNAseq FPKM log2-fold change > 0.4, and *q* < 0.05). Each region was further sorted into one of four possible combinations: (1) decreased methylation (diff. Methyl < 0) and decreased chromatin accessibility (log2-fold change fiber/epi < 0, and *q* < 0.05); (2) decreased methylation (diff. Methyl < 0) and increased chromatin accessibility (log2-fold change fiber/epi > 0, and *q* < 0.05); (3) increased methylation (diff. Methyl > 0) and decreased chromatin accessibility (log2-fold change fiber/epi < 0, and *q* < 0.05), or (4) increased methylation (diff. Methyl > 0) and increased chromatin accessibility (log2-fold change fiber/epi > 0, and *q* < 0.05). The resulting data can be found in Additional file 12: Table S8A, B and are visualized in Fig. 6A–D. Chi-square analysis was conducted to determine the potential association between differentially methylated mCG regions within differentially accessible chromatin regions at genebodies and promoters with corresponding gene expression changes between lens epithelial and fiber cells.

### Gene ontology and pathway analysis

The top 200 most highly expressed epithelial cell preferred genes (RNAseq FPKM log2FC < − 0.4, *q* < 0.05, ranked from highest to lowest FPKM in epithelial cells) and top 200 most highly expressed fiber cell preferred genes (RNAseq FPKM log2FC > 0.4, *q* < 0.05, ranked from highest to lowest FPKM in fiber cells) were each used as separate inputs to the Enrichr tool [52–54] to find overrepresented biological processes and pathways associated with lens differentiation state-specific genes. The resulting gene ontology biological processes and MSigDB [230] pathways are reported in Additional file 8: Table S4 and Additional file 2: Figure S2A–D.

The Enrichr tool was also used on the following groups of differentially expressed genes with at least one differentially methylated mCG region in the putative promoter or genebody: (1) fiber cell preferred genes (RNAseq FPKM log2FC > 0.4, *q* < 0.05) with an average diff.methyl < 0 of all differentially methylated mCG regions mapped to the promoter and genebody of the fiber cell preferred gene; (2) epithelial cell preferred genes (RNAseq FPKM log2FC < − 0.4, *q* < 0.05) with an average diff.methyl > 0 of all differentially methylated mCG regions mapped to the promoter and genebody of the epithelial cell preferred gene. The resulting overrepresented gene ontology biological processes and MsigDB pathways are reported in Additional file 10: Table S6A–D and Additional file 3: Figure S3A–D. The same analysis was also conducted for a smaller gene list containing only differentially expressed genes with differentially methylated promoters (Additional file 3: Figure S3E–H, Additional file 10: Table S6E–H).

### Transcription factor binding motif analysis

DNA sequences from differentially methylated mCG regions within the promoter (− 2 kb upstream from the transcription start site) and genebody of differentially expressed genes were analyzed for enriched transcription factor binding motifs using the AME tool from MEME-suite [65, 66]. The DNA sequences were separated into two groups: (1) sequences from demethylated mCG regions (diff.methyl < 0) of fiber cell preferred genes (RNAseq FPKM log2FC > 0.4, *q* < 0.05); (2) sequences from methylated mCG regions (diff.methyl > 0) of epithelial cell preferred genes (RNAseq FPKM log2FC < − 0.4, *q* < 0.05). Each group was analyzed separately with the AME tool versus a shuffled background according to default settings. The JASPAR nonredundant vertebrate transcription factor motif database [231] was used with the AME tool. All other default settings were kept. The resulting enriched transcription factor binding motifs were filtered to only include transcription factors with an average expression level of at least 1 FPKM as measured by RNAseq (Additional file 7: Table S3). Enriched transcription factor binding motifs for each group of sequences are in Additional file 13: Table S9 and visualized in Fig. 7A, B.

The MEME-suite FIMO tool [232] was used to identify the genomic coordinates of the enriched transcription factor binding sites identified by the AME tool using default settings. The JASPAR nonredundant vertebrate transcription factor motif database [231] was used as the motif input file. Target binding site methylation levels and corresponding gene expression levels for each predicted enriched transcription factor binding site are reported in Additional file 14: Table S10.

## Supplementary Information

**Additional file 1: Figure S1.** Additional comparisons of methylation levels between lens fiber cells and lens epithelial cells. **A** Pearson correlation analysis of biological triplicate samples of bisulfite sequenced genomic DNA from lens epithelial and fiber cells. **B** Dendrogram clustering of biological triplicate samples of bisulfite sequenced genomic DNA from lens epithelial and fiber cells. **C** Methylation levels (ratio of mCG/CG) at genomic regions within 2 kb of genebodies in lens epithelial and fiber cells. **D** Percent difference between the distribution of hypermethylated regions (more methylated in fiber cells) versus the distribution of hypomethylated regions (demethylated in fiber cells) at different genomic regions. Positive values indicate the corresponding genomic region contains a greater percentage of all hypermethylated regions than the percentage of all hypomethylated regions. Negative values indicate the inverse.

**Additional file 2: Figure S2.** Significant pathways and biological processes associated with differentially expressed genes between lens fiber and epithelial cells. **A** Top 10 GO Biological processes associated with the top 200 most highly expressed epithelial cell genes (RNAseq log2FC < − 0.4, adjusted *p* < 0.05, ranked from most to least FPKM in epithelial cells). **B** Top 10 MSigDB Hallmark pathways. Same gene set as **A**. **C** Top 10 GO Biological processes associated with the top 200 most highly expressed fiber cell genes (RNAseq log2FC > 0.4, adjusted *p* < 0.05, ranked from most to least FPKM in fiber cells). **D** Top 10 MSigDB Hallmark pathways. Same gene set as **B**.

**Additional file 3: Figure S3.** Significant pathways and biological processes associated with DEGs with DMRs between lens fiber and epithelial cells. **A** Top 10 MSigDB Hallmark pathways associated with upregulated/fiber cell genes (RNAseq log2FC > 0.4, adjusted *p* < 0.05) that also have decreased average methylation levels at DMRs in the promoter and genebody. Blue colored data indicate a statistically significant association adjusted *p* < 0.05. **B** Top 10 GO Biological processes. Same gene set as **A**. **C** Top 10 MSigDB Hallmark pathways associated with downregulated/epithelial cell genes (RNAseq log2FC < − 0.4, adjusted *p* < 0.05) that also have increased average methylation levels at DMRs in the promoter and genebody. Blue colored data indicate a statistically significant association adjusted *p* < 0.05. **D** Top 10 GO Biological processes. Same gene set as **C**. **E** Top 10 MSigDB Hallmark pathways associated with upregulated/fiber cell genes (RNAseq log2FC > 0.4, adjusted *p* < 0.05) that also have decreased average methylation levels at DMRs only in the promoter. Blue colored data indicate a statistically significant association adjusted *p* < 0.05. **F** Top 10 GO Biological processes. Same gene set as **E**. **G** Top 10 MSigDB Hallmark pathways associated with downregulated/epithelial cell genes (RNAseq log2FC < − 0.4, adjusted *p* < 0.05) that also have increased average methylation levels at DMRs only in the promoter. Blue colored data indicate a statistically significant association adjusted *p* < 0.05. **H** Top 10 GO Biological processes. Same gene set as **G**.

**Additional file 4: Figure S4.** Most chromatin-accessible regions do not contain differentially methylated regions. Proportion of chromatin-accessible regions that contain differentially methylated regions or no significant changes in methylation levels. Opening chromatin refers to regions with ATACseq log2FC > 0, adj. *p* < 0.05. Closing chromatin refers to regions with ATACseq log2FC < 0, adj. *p* < 0.05. Stable/unchanged chromatin, ATACseq adj. *p* > 0.05.

**Additional file 5: Table S1.** Proportion of methylated cytosines that are mCG, mCHG, mCHH in each sample of lens epithelial and fiber cells.

**Additional file 6: Table S2.** Differentially methylated mCG regions between lens epithelial and fiber cells.

**Additional file 7: Table S3.** Differentially expressed genes between lens epithelial cells and fiber cells. **A** Epithelial preferred genes (FPKM log2FC < − 0.4, *q* < 0.05), **B** Fiber preferred genes (FPKM log2FC > 0.4, *q* < 0.05). **C** Non-differentially expressed genes.

**Additional file 8: Table S4.** Enrichr outputs of GO Biological processes (**A**, **C**) and MSigDB Hallmark pathways (**B**, **D**) significantly associated with the differentially expressed genes between lens epithelial cells and fiber cells. **A**, **B** Top 200 epithelial cell preferred genes, **C**, **D** top 200 fiber cell preferred genes.

**Additional file 9: Table S5.** Average change in mCG methylation level for all differentially methylated mCG regions mapped to **A**–**D** the promoters and genebodies of differentially expressed genes, or **E**–**H** only the promoters of differentially expressed genes. **A**, **E** Epithelial cell preferred genes with increased mCG methylation. **B**, **F** Fiber cell preferred genes with decreased mCG methylation. **C**, **G** Epithelial cell preferred genes with decreased mCG methylation. **D**, **H** Fiber cell preferred genes with increased mCG methylation.

**Additional file 10: Table S6.** Enrichr outputs of MSigDB Hallmark pathways (**A**, **C**, **E**, **G**) and GO biological processes (**B**, **D**, **F**, **H**) significantly associated with differentially expressed genes containing differentially methylated promoters and/or gene bodies. **A**, **B** Epithelial cell preferred genes with increased average promoter and genebody mCG methylation levels in fiber cells. **C**, **D** Fiber cell preferred genes with decreased average promoter and genebody mCG methylation levels in fiber cells. **E**, **F** Epithelial cell preferred genes with increased average promoter mCG methylation levels in fiber cells. **G**, **H** Fiber cell preferred genes with decreased average promoter mCG methylation levels in fiber cells.

**Additional file 11: Table S7.** Differentially methylated mCG regions that are at least partially contained within open chromatin regions.

**Additional file 12: Table S8.** Differentially methylated mCG regions that are at least partially contained within open chromatin regions at **A** promoters and genebodies of differentially expressed genes, or **B** only promoters of differentially expressed genes.

**Additional file 13: Table S9.** Significantly enriched transcription factor binding motifs found within **A** hypermethylated mCG regions in promoters or genebodies of epithelial cell preferred genes, or **B** hypomethylated mCG regions in promoters or genebodies of epithelial cell preferred genes.

**Additional file 14: Table S10.** Target binding sites of enriched transcription factor binding motifs along with corresponding methylation changes and gene expression changes. Target binding sites of enriched transcription factor motifs from demethylated DMRs of Fiber cell genes (**A**), and from methylated DMRs of Epithelial cell genes (**B**).

**Additional file 15: Table S11.** Genes with established lens functions or associations with cataracts that are differentially expressed and contain differentially methylated regions between lens epithelial and fiber cells.

**Additional file 16: Table S12.** Pearson correlation analysis comparing gene expression changes and methylation changes within open chromatin regions. The analyses were performed on the following conditions: differentially methylated regions at (1) promoters and genebodies, (2) only promoters, (3) open and closed chromatin, (4) only open chromatin, (5) only open chromatin with significant changes in chromatin accessibility between lens epithelial and fiber cells (ATACseq adj.*p*-val < 0.05).

## Data Availability

Raw sequencing reads for the whole genome bisulfite experiment have been deposited at NCBI-GEO series accession number GSE196629. Raw sequencing reads for the RNA-seq experiment have been deposited at NCBI-GEO series accession number GSE196630. ATACseq data were obtained from supplementary files from [32].

## Abbreviations

ATAC-seq: Assay for transposase-accessible chromatin sequencing
TSS: Transcription start site
FPKM: Fragments per kilobase of exon per million fragments mapped
DNMTs: DNA methyltransferases
DMR: Differentially methylated region
DEG: Differentially expressed gene
