# Supplementary figures and images for: Changes in DNA methylation hallmark alterations in chromatin accessibility and gene expression for eye lens differentiation
Source: Epigenetics Chromatin. 2022 Mar 5;15:8. doi: 10.1186/s13072-022-00440-z (PMC8897925; doi:10.1186/s13072-022-00440-z)

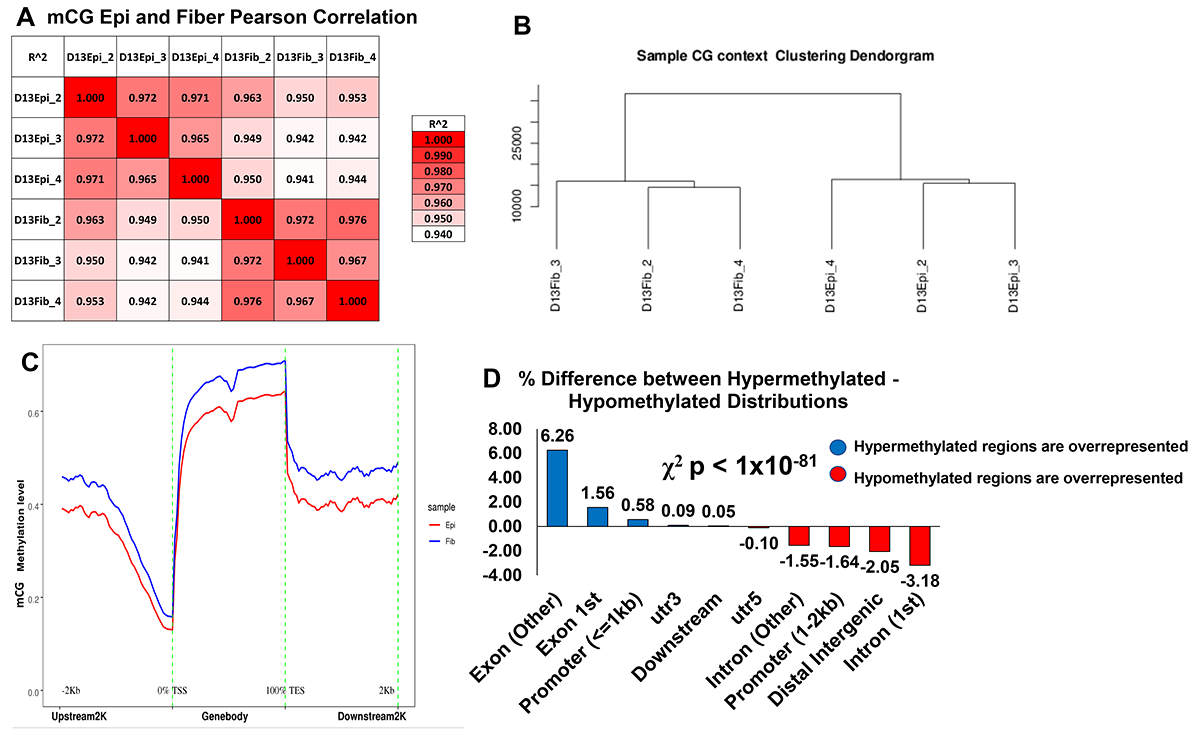

Supplement: Supplementary file 1 — Additional file 1: Figure S1. Additional comparisons of methylation levels between lens fiber cells and lens epithelial cells. A Pearson correlation analysis of biological triplicate samples of bisulfite sequenced genomic DNA from lens epithelial and fiber cells. B Dendrogram clustering of biological triplicate samples of bisulfite sequenced genomic DNA from lens epithelial and fiber cells. C Methylation levels (ratio of mCG/CG) at genomic regions within 2 kb of genebodies in lens epithelial and fiber cells. D Percent difference between the distribution of hypermethylated regions (more methylated in fiber cells) versus the distribution of hypomethylated regions (demethylated in fiber cells) at different genomic regions. Positive values indicate the corresponding genomic region contains a greater percentage of all hypermethylated regions than the percentage of all hypomethylated regions. Negative values indicate the inverse. [file 13072_2022_440_MOESM1_ESM.tif]

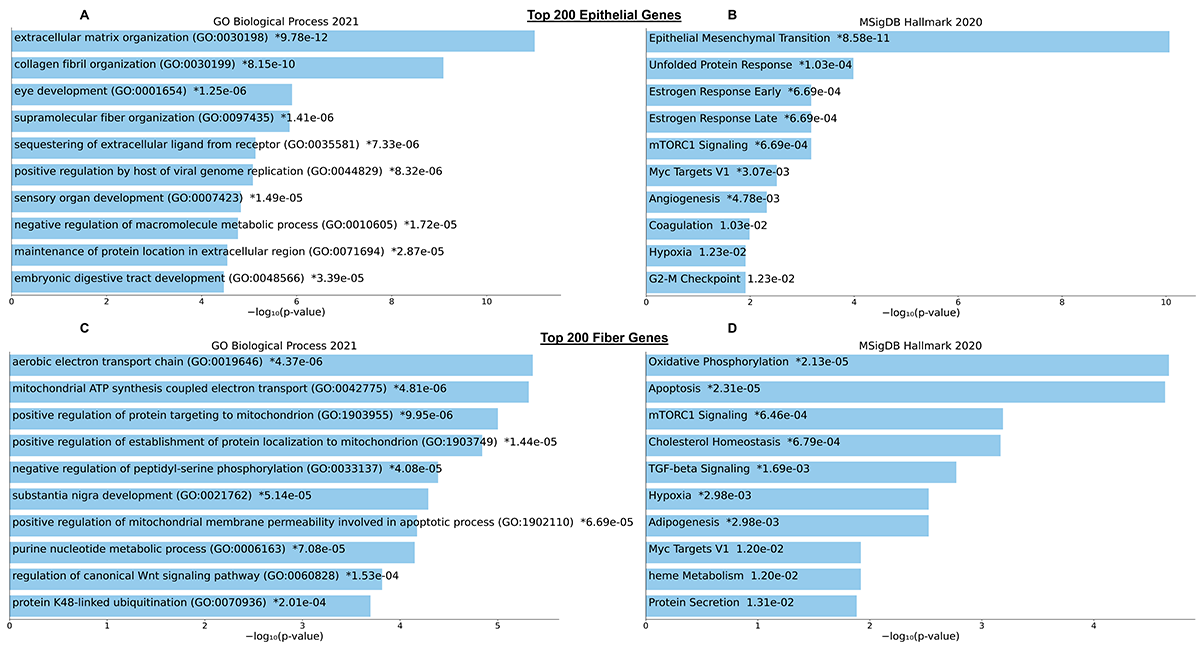

Supplement: Supplementary file 2 — Additional file 2: Figure S2. Significant pathways and biological processes associated with differentially expressed genes between lens fiber and epithelial cells. A Top 10 GO Biological processes associated with the top 200 most highly expressed epithelial cell genes (RNAseq log2FC < − 0.4, adjusted p < 0.05, ranked from most to least FPKM in epithelial cells). B Top 10 MSigDB Hallmark pathways. Same gene set as A. C Top 10 GO Biological processes associated with the top 200 most highly expressed fiber cell genes (RNAseq log2FC > 0.4, adjusted p < 0.05, ranked from most to least FPKM in fiber cells). D Top 10 MSigDB Hallmark pathways. Same gene set as B. [file 13072_2022_440_MOESM2_ESM.tif]

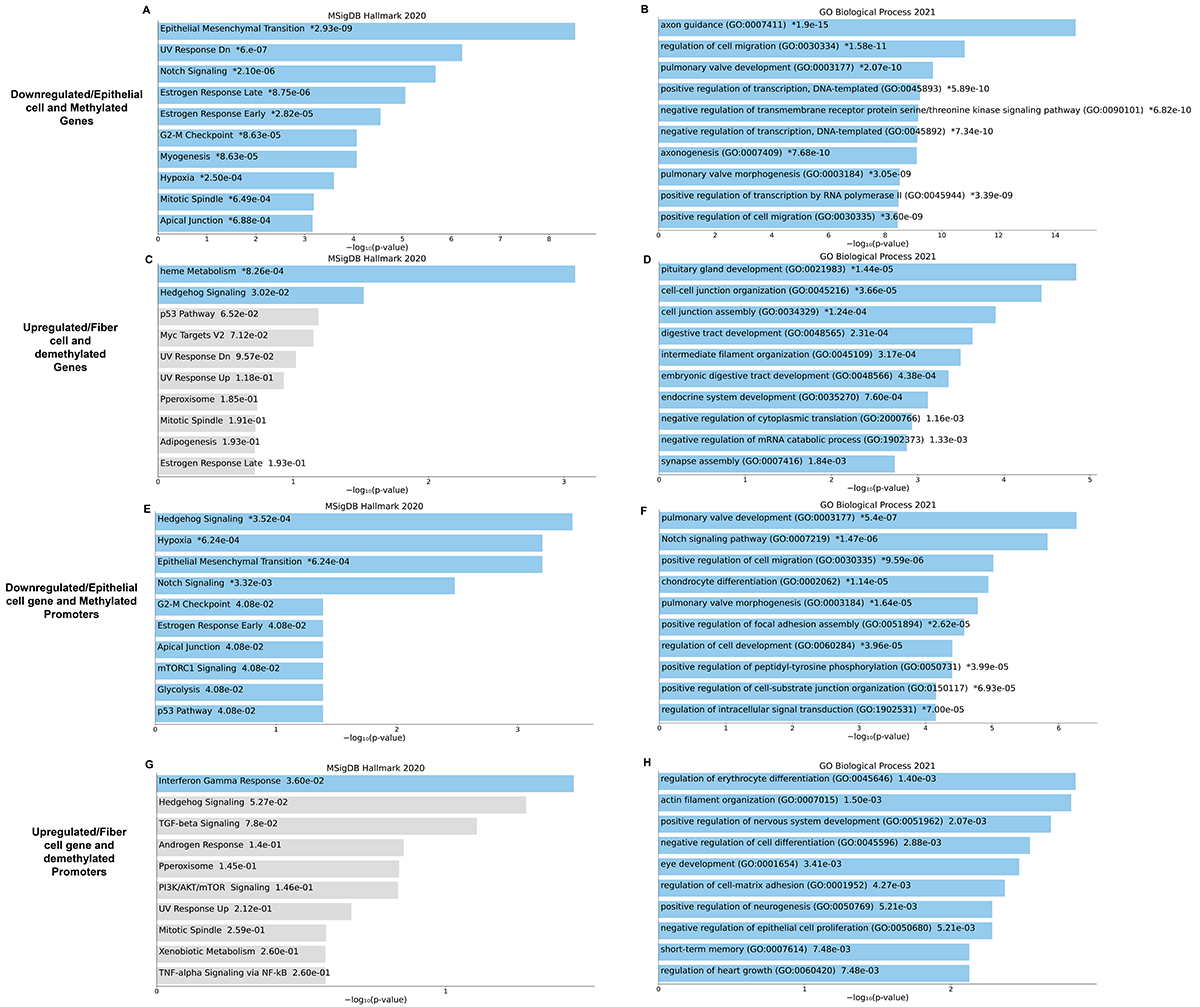

Supplement: Supplementary file 3 — Additional file 3: Figure S3. Significant pathways and biological processes associated with DEGs with DMRs between lens fiber and epithelial cells. A Top 10 MSigDB Hallmark pathways associated with upregulated/fiber cell genes (RNAseq log2FC > 0.4, adjusted p < 0.05) that also have decreased average methylation levels at DMRs in the promoter and genebody. Blue colored data indicate a statistically significant association adjusted p < 0.05. B Top 10 GO Biological processes. Same gene set as A. C Top 10 MSigDB Hallmark pathways associated with downregulated/epithelial cell genes (RNAseq log2FC < − 0.4, adjusted p < 0.05) that also have increased average methylation levels at DMRs in the promoter and genebody. Blue colored data indicate a statistically significant association adjusted p < 0.05. D Top 10 GO Biological processes. Same gene set as C. E Top 10 MSigDB Hallmark pathways associated with upregulated/fiber cell genes (RNAseq log2FC > 0.4, adjusted p < 0.05) that also have decreased average methylation levels at DMRs only in the promoter. Blue colored data indicate a statistically significant association adjusted p < 0.05. F Top 10 GO Biological processes. Same gene set as E. G Top 10 MSigDB Hallmark pathways associated with downregulated/epithelial cell genes (RNAseq log2FC < − 0.4, adjusted p < 0.05) that also have increased average methylation levels at DMRs only in the promoter. Blue colored data indicate a statistically significant association adjusted p < 0.05. H Top 10 GO Biological processes. Same gene set as G. [file 13072_2022_440_MOESM3_ESM.tif]

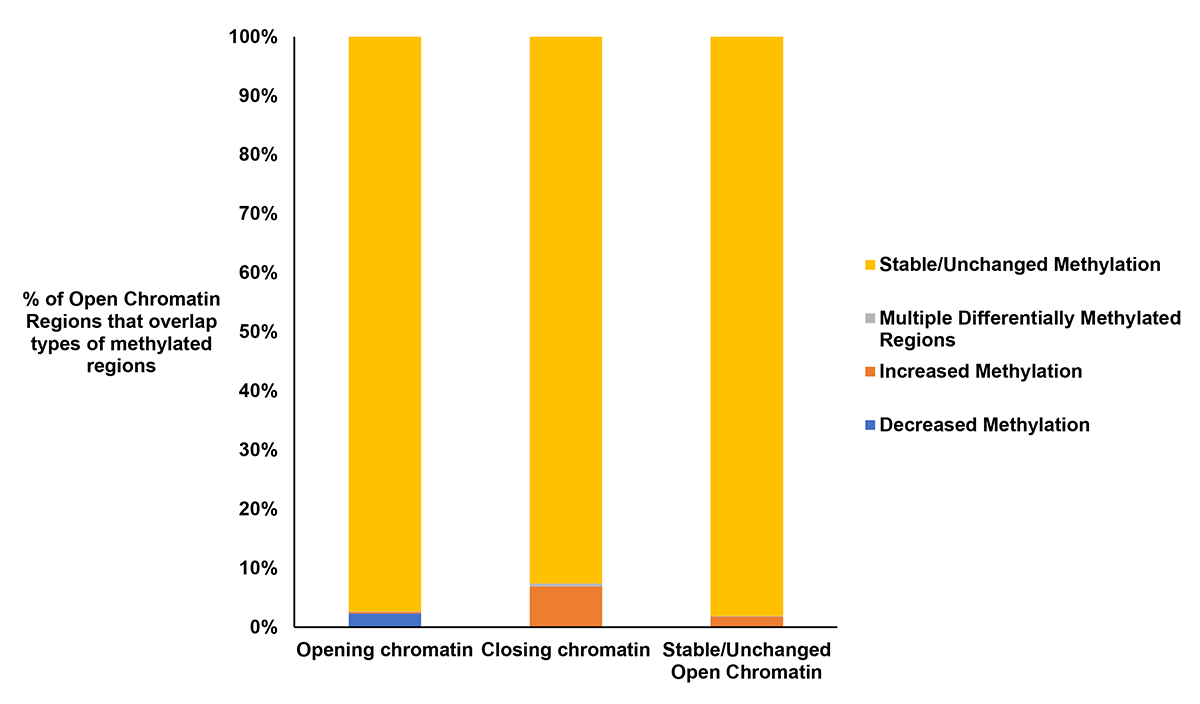

Supplement: Supplementary file 4 — Additional file 4: Figure S4. Most chromatin-accessible regions do not contain differentially methylated regions. Proportion of chromatin-accessible regions that contain differentially methylated regions or no significant changes in methylation levels. Opening chromatin refers to regions with ATACseq log2FC > 0, adj. p < 0.05. Closing chromatin refers to regions with ATACseq log2FC < 0, adj. p < 0.05. Stable/unchanged chromatin, ATACseq adj. p > 0.05. [file 13072_2022_440_MOESM4_ESM.tif]
